# *MYB1R1* and *MYC2* Regulate ω-3 Fatty Acid Desaturase Involved in ABA-Mediated Suberization in the Russet Skin of a Mutant of ‘Dangshansuli’ (*Pyrus bretschneideri* Rehd.)

**DOI:** 10.3389/fpls.2022.910938

**Published:** 2022-06-09

**Authors:** Qi Wang, Yaping Liu, Xinyi Wu, Lindu Wang, Jinchao Li, Minchen Wan, Bin Jia, Zhenfeng Ye, Lun Liu, Xiaomei Tang, Shutian Tao, Liwu Zhu, Wei Heng

**Affiliations:** ^1^College of Horticulture, Anhui Agricultural University, Hefei, China; ^2^College of Horticulture, Nanjing Agricultural University, Nanjing, China

**Keywords:** pear, russet skin, suberization, abscisic acid, fatty acid desaturase, transcription factor

## Abstract

Russeting, a disorder of pear fruit skin, is mainly caused by suberin accumulation on the inner part of the outer epidermal cell layers. ABA was identified as a crucial phytohormone in suberification. Here, we demonstrated that the ABA content in russet pear skin was higher than in green skin. Then, ABA was applied to explore the changes in phenotype and suberin composition coupled with RNA-Seq and metabolomics to investigate the probably regulatory pathway of ABA-mediated suberification. The results showed that ABA treatment increased the expression of ω-3 fatty acid desaturase (FAD) and the content of α-linolenic acid. We identified 17 PbFADs in white pear, and the expression of *PbFAD3a* was induced by ABA. In addition, the role of *PbFAD3a* in promoting suberification has been demonstrated by overexpression in Arabidopsis and VIGS assays in the fruitlets. GUS staining indicated that the promoter of *PbFAD3a* was activated by ABA. Furthermore, *MYC2* and *MYB1R1* have been shown to bind to the *PbFAD3a* promoter directly and this was induced by ABA via yeast one-hybrid (Y1H) screening and qRT–PCR. In summary, our study found that ABA induces the expression of *MYC2* and *MYB1R1* and activates the *PbFAD3a* promoter, contributing to the formation of russet pear skin. Functional identification of key transcription factors will be the goal of future research. These findings reveal the molecular mechanism of ABA-mediated suberization in the russet skin and provide a good foundation for future studies on the formation of russet skin.

## Introduction

Pear (*Pyrus* spp.), one of the most economically important temperate tree fruit crops belonging to the Rosaceae family, has been cultivated by humans for more than 3,000 years (Rom and Carlson, 1987). Fruit color is an especially important index to measure its appearance quality and is a critical factor affecting its commercial value. Pear fruits can be classified into green, full russet, partial russet, and red groups based on their skin color (Inoue et al., 2006; Charoenchongsuk et al., 2018). ‘Dangshansuli’ (*Pyrus bretschneideri* Rehd.) belongs to the green-skin groups, accounting for the largest proportion of pear fruits in China. A mutant of ‘Dangshansuli’, showing a reddish-brown color with full russeting, was investigated in 2001 and named ‘Dangshanjinsu’ (Heng et al., 2014). However, the gene regulatory network of russet fruit skin formation remains largely unknown.

So far, the molecular mechanisms of russet skin formation in sand pear and apple have been investigated by excavating the key pathways and genes specific to skin russeting (Legay et al., 2016; Wang et al., 2020; Zhang et al., 2021). The tissue of pear fruit skin is mainly comprised of the cuticle, epidermal cells, and suberin meristem (Wang et al., 2020). The specialized epidermal tissues of plants, which are covered by the cuticle, an extracellular layer, play a crucial role in protecting against and resisting abiotic stress (Petit et al., 2014).

Suberin and cutin are derivatives of fatty acid polymers connected by ester bonds called polyester (Beisson et al., 2012), which is a self-protection mechanism of plants that was gradually formed during long term evolution in response to stress (Pollard et al., 2008; Petit et al., 2014). In most species, cutin is mainly comprised of C16 and C18 ω-hydroxy fatty acids (Pollard et al., 2008). Distinctively in suberin, it is constituted by C16 to C26 aliphatic monomers, including ω-hydroxy acids, α,ω-diacids, primary fatty alcohols, and high levels of glycerol (Pollard et al., 2008). Studies have shown that suberin contains two major domains, suberin polyphenolics and polyaliphatics (Kolattukudy, 1984; Bernards, 2002; Dastmalchi et al., 2015). Polyphenolics are synthesized via the phenylpropanoid biosynthesis pathway (PPP) and mainly consist of cinnamic acid, p-coumaric acid, ferulic acid, and their derivatives (Graa and Santos, 2007). Polyaliphatics are comprised of fatty acids, α,ω-diacids, long-chain ω-hydroxyacids, alcohols, alkanes, dimeric esters, and glycerol (Rocha et al., 2001; Wang et al., 2010; Tao et al., 2016). The polyphenolics synthesized from the PPP and polyaliphatics produced by lipid metabolism have a certain connection and together contribute to suberin formation (Bernards, 2002; Serra et al., 2010; Ranathunge et al., 2011).

Abscisic acid (ABA) is a hormone involved in major plant responses to stress, such as pathogens, salt, temperature, mechanical damage, and water scarcity (Mizrahi and Richmond, 1970; Savchenko et al., 2014). Barberon et al. found that suberin development in plant roots are controlled by ABA (Barberon et al., 2016). To date, ABA-mediated suberization has been demonstrated in Arabidopsis (Efetova et al., 2007; Barberon et al., 2016; Shukla et al., 2021), tomato (Leide et al., 2012; Tao et al., 2016), kiwifruit (Han et al., 2018; Wei et al., 2020a), and potato (Kumar et al., 2010; Pau et al., 2013). Russeting of pear exocarp has been identified to be caused by the rupture of the outermost cells on the fruit surface, forming microscopic cracks when the tension exceeds the capacity of the epidermis and then forming a membrane-like layer of suberin (Khanal et al., 2013; Kwon et al., 2016). Hence, we can consider russet formation as a wound-healing process. To date, the function of ABA in the formation of russet pear skin has remained obscure. The purpose of this study was to characterize the molecular mechanism of ABA-mediated suberization and provide a novel elucidation of how ABA stimulates russet skin formation.

## Materials and Methods

### Plant Materials

The plant materials were obtained from the Horticulture Farm of Dangshan County, Anhui Province during the growing season in 2019. Typical ‘Dangshansuli’ and ‘Dangshanjinsu’ plants were selected to ensure the rigor of the experiment and the consistency of the fruit development background. A total of 120 fruits of uniform size and growing positions were selected for treatments, including deionized water control and ABA (100 μM, Sigma A1049) at 10, 25, 50, 75, 100, 125, 150, and 175 days after full bloom (DAFB). The fruits were soaked in the reagent for 30 s. The sampling time was 25, 100, and 175 DAFB. The exocarp was dissected with a double-sided blade from the fruit skin, immediately placed in liquid nitrogen, and stored at −80 °C.

### Determination of Endogenous ABA Content

After grinding, 1 g of fresh plant sample was mixed with 10 ml isopropanol/hydrochloric acid extraction buffer and shaken for 30 min at 4 °C. Then, 20 ml of dichloromethane was added and it was shaken for 30 min at 4 °C. The lower organic phase was extracted after centrifugation at 13,000 rpm/min at 4 °C. After drying with nitrogen, the solution was dissolved in 400 μl methanol solution containing 0.1% formic acid. After 0.22 m filtration, a preparation was made for HPLC–MS/MS detection. A Qtrap6500 mass spectrometer (Applied Biosystems, Foster City, California) was used in this experiment. A Methanol solution containing 0.1% formic acid was used to prepare an ABA standard solution with a concentration gradient (0.1, 0.2, 0.5, 2, 5, 20, 50, and 200 ng/mL). The bad linear points were removed in the actual drawing of the standard curve equation.

### Paraffin Section Analysis of Pear Exocarp

After cleaning the pear fruit surface, a sample of the pear exocarp measuring 0.5 cm × 0.7 cm was removed using a double-sided blade and fixed in FAA solution (acetic acid:formalin:water:95% ethanol mixed at a volume ratio of 1:3:7:10) for more than 24 h. It was then dehydrated with gradient alcohol. Then, the tissue was embedded in paraffin wax. After cooling to −20 °C, the tissue block was sliced to a thickness of approximately 4 μm with a paraffin slicer. The tissue slices were baked in an oven at 60 °C. Safranin O staining solution was used for staining and rapid dehydration with anhydrous ethanol. Then, the sections were placed into plant solid green staining solution for 6~20 s and rapidly dehydrated with anhydrous ethanol. Finally, neutral balsam was used to mount the tissue sections, which were observed under a microscope, and images were taken.

### Measurement of Suberin Monomers in the Exocarp of Pear

Two grams of pear exocarp was subjected to cellulase and pectinase for hydrolysis. The samples were dried at 50°C. After extraction, the solids were cleaned with acetone. After drying, soluble lipids were removed with organic solvent extraction, and a methanol solution containing 14% BF_3_ (trifluoro(methanol)boron, CH_4_BF_3_O) was used for depolymerization for 2 h to obtain the final lipid components. The final lipid components were extracted with chloroform. Then, triacontane (100 μg) was added as an internal standard. The derivatization process was conducted by the addition of 150 μl N, O-bis-(trimethylsilyl) trifluoroacetamide (BSTFA) at 70°C for 40 min. Residues were prepared for gas chromatography-mass (GC–MS) analysis via an Agilent 7890A GC/5975C MS.

### RNA Extraction and Sequencing

Total RNA was extracted from pear fruit skin via the CTAB method (Jordon-Thaden et al., 2015). A BGISeq500 platform (BGI-Shenzhen, China) was used to generate single-end 50-base reads. Before data analysis, low-quality reads and connector contamination, coupled with the proportion of N > 5%, were removed to guarantee the dependability of the data. Then, the clean reads were mapped to the reference genome of *Pyrus bretschneideri* (Wu et al., 2013) using HISAT (Kim et al., 2015). Clean reads were compared to the reference gene sequence using Bowtie2 (Langmead and Salzberg, 2012), and then RSEM (Dewey and Li, 2011) was used to calculate the gene expression levels. Transcripts were assembled and annotated from the read alignment results using Cufflinks v2.1.1 (Trapnell et al., 2012).

### Differential Expression Analysis

The accession number for the transcriptome data was PRJNA807059. The expression level of the genes was calculated based on the fragments per kilobase of transcript per million mapped reads (FPKM) (Likun et al., 2010). All successfully mapped reads were subjected to differential expression analysis using DESeq2 (Love et al., 2014). Transcripts with fold change (FC) > −2 (upregulated) or < −2 (downregulated) and with an adjusted *p*<0.001 were considered significant. Annotation and enrichment analysis was based on the KEGG database (Kyoto Encyclopedia of Genes and Genomes, http://www.genome.jp/kegg/). The Phyper function in R software was used to classify the differentially expressed genes into biological pathways based on the KEGG annotation results and official classification.

### Metabolite Extraction and Analysis

Data acquisition was performed using an advanced Xevo G2-XS QTOF mass spectrometer (Waters, UK)(Pope et al., 2017). The raw data were imported into Progenesis QI (Waters, UK) for peak alignment, picking, and identification (Dunn et al., 2011). Data preprocessing and filtering out ions with a relative standard deviation (RSD) >30% were performed using metaX (Wen et al., 2017). Identification and annotation were based on the KEGG database (Kanehisa and Goto, 2000). This project used variable importance in projection values of the first two principal components in the multivariate PLS-DA model, combined with fold change (FC) and q-values from a univariate analysis to choose differential metabolites (variable importance in projection values ≥ 1 and FC ≥ 1.2 or ≤ 0.833 and with an adjusted q-value < 0.05, all three must be met for an ion to be considered as a differential ion).

### Identification and Phylogenetic Analysis of PbFADs

The PF00487 domain model files of PbFAD family members were downloaded from the PFAM website (https://www.pfam.org). The candidate genes containing PF00487 domains were identified (E-value=e^−10^) using HMMER v.3.2 (Yap et al., 2016). The FAD protein sequences of white pear and Arabidopsis were extracted and aligned. The InterProScan program was used on all of the candidate protein pairs and confirmed the presence of the diagnostic domain using the Pfam and SMART databases. MAFFT used default parameters to align the multiple homologous FAD genes. A phylogenetic tree was constructed using the maximum likelihood method [(bootstraps = 1,000) and IQ-TREE 1.6.9 sofware (Yap et al., 2016)].

### Gene Cloning and Arabidopsis Transformation

The 1,362 bp coding sequence of *PbFAD3a* (*Pbr021630*.1) was cloned via PCR using primer pairs containing restriction sites *XbaI* and *BamHI* using a cloning kit provided by KOD FX Neo (TOYOBO, Shanghai). The coding sequence of *PbFAD3a* was inserted into the pCAMBIA1300 vector by the homologous recombination method to generate the fusion construct p35S-PbFAD3a. The plasmids were extracted by the plasmid extraction kit provided by Vazyme Biotech (Nanjing, China). Then, the fusion constructs p35S-PbFAD3a was transferred to GV3101 cells via the freeze–thaw method (Weigel and Glazebrook, 2006). *Agrobacterium*-mediated genetic transformation of Arabidopsis was performed, and T0 generation transgenic plants overexpressing *PbFAD3a* were obtained. Via the use of antibiotics (hygromycin B, 25 mg/L) for three generations of seed screening, T3 transgenic lines were obtained.

### Subculture of Pear Calli

The flesh of European pear (*Pyrus communis*) fruitlets was used to induce pear calli as previously described (Bai et al., 2019). The subculture of pear calli was conducted as previously described (Wang et al., 2022). In brief, the pear calli were cultured on solid MS medium supplemented with 1 mg/L 2,4-D and 0.5 mg/L 6-BA at 25 °C in the dark. For the ABA treatments, pear calli were cut into 5–7 mm pieces, and 18 petri dishes of calli were used. The pear calli of the same size were treated in the same petri dish were considered as one biological replicate, and analyses were completed with at least three biological replicates.

### VIGS Assays

The ‘Xiangnan’ pear (*Pyrus pyrifolia*) fruitlets (35 DAFB) obtained from the Fruit and Tea Research Institute, Hubei Academy of Agricultural Sciences were used for the VIGS assays. It can achieve a full russet at 50 DAFB, making it a good material for the analysis of russet pear skin. A *PbFAD3a* fragment (1-396 bp) was amplified using the primer pair pTRV2-PbFAD3a-F/pTRV2-PbFAD3a-R and cloned into the pTRV2 vector (pTRV2-PbFAD3a). Then, the fusion vector pTRV2-PbFAD3a was imported into *Agrobacterium* strain GV3101 and used for pear fruit skin infection. Agrobacterium cells were resuspended in the infection solution (98 ml deionized water + 1 ml 1 M MgCl_2_ + 1 ml 1 M MES + 200 μl 200 mM acetosyringone, pH 5.6). The injection solution harboring pTRV2-PbFAD3a, pTRV2-pTRV1 (negative control), and pure water were coinfiltrated into the fruitlets exocarp of ‘Xiangnan’ pear on the tree. The fruit exocarp was slowly injected until the water stain was 2 cm in diameter. Then, 14 days after infiltration, the samples were collected.

### Promoter Activity Analysis

The expression vector comprised of the GUS (β-glucuronidase reporter gene) coding sequence driven by the *PbFAD3a* promoter was constructed by introducing the *PbFAD3a* promoter into the *BamHI* and *EcoRI* cloning sites of the pCAMBIA1391::GUS vector using the corresponding primers (Supplementary Table S5). The fusion vector was introduced into *Agrobacterium* strain GV3101 and then used for tobacco leaf infiltration. Three days after injection, the injected leaves were cut and soaked in GUS staining solution (10 mM EDTA+100 mM Na_3_PO_4_.12H_2_O + 0.5 mM K_4_Fe(CN)_6_·H_2_O + 2 mM X-gluc +0.1% Triton X-100) for 36 h. Then, ethanol was used for decolorization. GUS activity was measured using a GUS gene quantitative detection kit (Coolaber, Beijing).

### qRT-PCR Verification

A TRIzol kit provided by Tiangen Biotech (Beijing) was used to extract total RNA from pear skin. A cDNA Reverse Transcription Kit provided by Takara (Beijing) was used to synthesize single-stranded cDNA. Quantitative real-time PCR (qRT–PCR) was performed using the SYBR^®^ Green Premix kit (Toyobo, Shanghai) in an ABI Step-one Plus PRISM 7300 System (Applied Biosystems, Foster City, California). The reaction systems and procedures were performed according to the methods described by Heng et al. (2016). The 2^−ΔΔCT^ method was used to calculate the relative expression levels (Livak and Schmittgen, 2001).

### Yeast One-Hybrid Assays

The *PbFAD3a* promoter (1,875 bp) was inserted into a pAbAi vector. Then, the fusion construct was introduced into yeast strain Y1H-GOLD. Self-activation was tested on SD medium without Ura (SD-Ura) containing 0, 50, 100, 200, and 300 ng/mL aureobasidin A (AbA). Y1H screening was conducted using pAbAi-pro-PbFAD3a cells as bait. The russet and green pear exocarp cDNA library was transformed into bait yeast cells and plated on freshly prepared SD-Trp + 300 ng/ml AbA plates. Positive clones were selected and transferred to SD-Trp + 300 ng/mL AbA plates twice and then sequenced.

In the interaction tests, the coding sequences of *PbMYC2* (*Pbr037113.1*) and *PbMYB1R1* (*Pbr011534.1*) were cloned and integrated into the pGADT7 (AD) prey vector (generating the fusion constructions AD-PbMYC2 and AD- PbMYB1R1) and then transferred into individual bait-reporter yeast strains. The transformed Y1HGold plants were cultivated in SD medium with 300 ng/ml AbA and without leucine (SD-Leu+AbA^300^) at 28°C for 3 d to test the interactions. pGADT7-53 (AD-53) was cotransformed with pAbAi-p53 as a positive control, and AD-empty and pro-PbFAD3a-AbAi were transformed as negative controls.

### Dual Luciferase Assays

Full-length *PbMYC2* (*Pbr037113.1*) and *PbMYB1R1* (*Pbr011534.1*) were individually inserted into the pGreen II 0029 62-SK vector (SK), and 1,875 bp of the *PbFAD3a* promoter was inserted into the pGreen II 0800-LUC vector. All of the constructs were transformed into the GV3101 strain via the freeze–thaw method (Weigel and Glazebrook, 2006). Dual luciferase assays were conducted on *N. benthamiana* grown for 5–6 weeks in a suitable environment. The infected *Agrobacterium* liquid was prepared with 10 mM MgCl_2_, 10 mM MES, and 200 μM acetosyringone (OD_600_ = 0.8). The mixtures of infiltration buffer contained two TFs and the *PbFAD3a* promoter at the volume ratio of 9:1. Then, needleless syringes were used to inject the mixtures into the abaxial side of the leaves. The leaves were sampled 2–3 d after injection, and Firefly luciferase and Renilla luciferase were analyzed using a Dual-Luciferase Reporter Assay System (Promega). Three independent experiments with six replications each were conducted.

## Results

### ABA Plays a Crucial Role in Russet Pear Skin Formation

**Figure 1:**
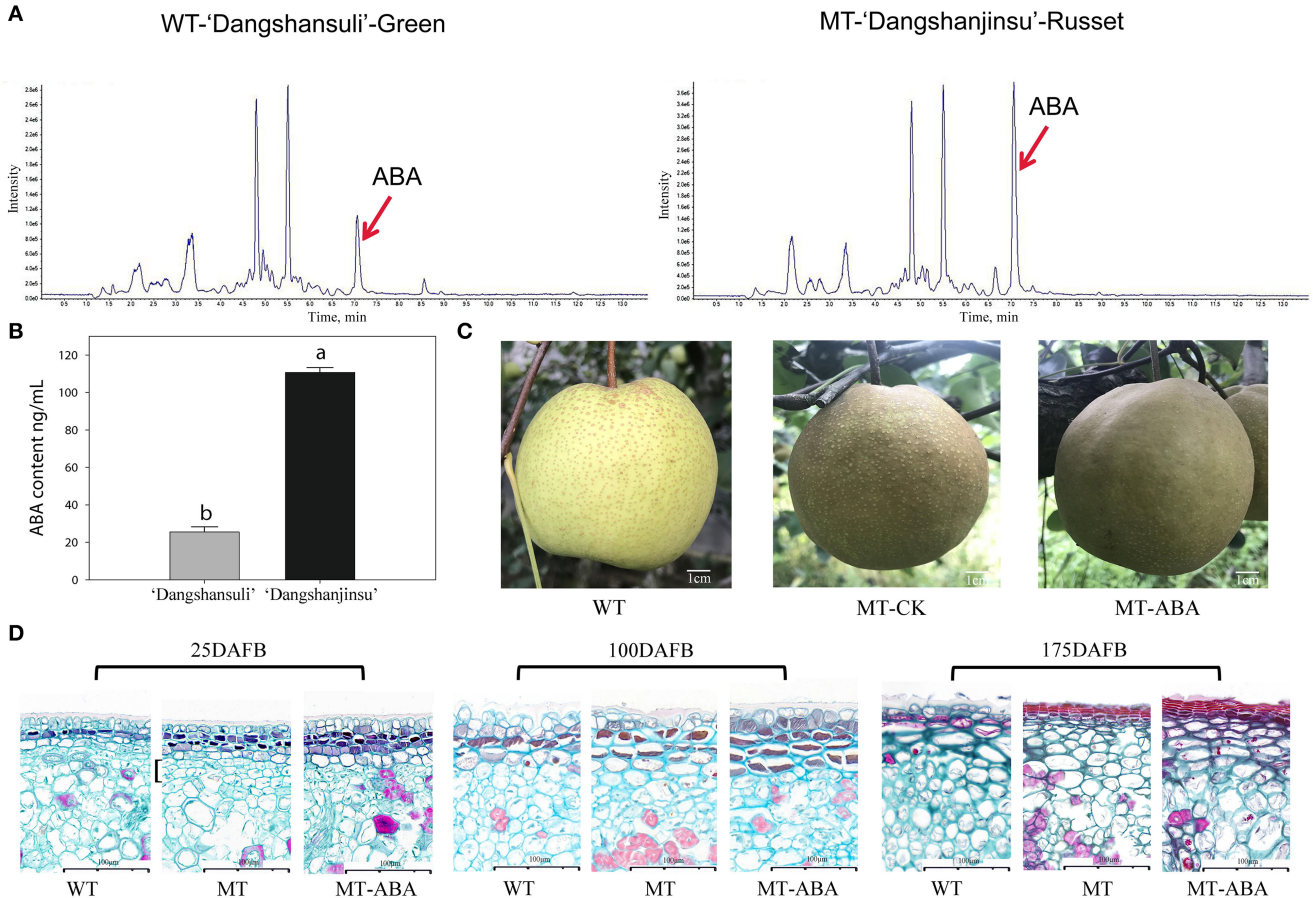
ABA is involved in russet pear skin formation. **(A,B)** ESI-HPLC-MS/MS analysis of ABA content in russet and green pear skin. **(C)** Phenotype of ‘Dangshansuli’, ‘Dangshanjinsu’ and ‘Dangshanjinsu’ treated with ABA at 175DAFB. **(D)** Paraffin slices observation of pear exocarp. Cross section stained with Safranin O-Fast green. The suberized, lignified, and fibrotic cell walls are stained with reddish brown, red, and green, respectively. WT, ‘Dangshansuli’. MT, ‘Dangshanjinsu’. DAFB, days after full bloom. Bars = 100 μm. The error bars are the means ± SD of three biological repeats and lowercase letters indicate significant differences by two-tailed Student's *t*-test (*p* < 0.01).

To analyze the role of endogenous ABA signaling characteristics in the formation of russet fruit skin, the content of ABA between ‘Dangshansuli’ and ‘Dangshanjinsu’ was determined by ESI-HPLC–MS/MS. The results showed that the ABA content of russet skin was significantly higher than that of green skin (Figure 1A). The average concentrations of the three biological replicates of endogenous ABA in green and russet fruit skin were 25.48 and 110.63 ng/g, respectively (Figure 1B). In addition, we found that the application of exogenous ABA increased the suberification and russeting of ‘Dangshanjinsu’ exocarp (Figure 1C). The epidermal cells of pear exocarp began to lignify at 100 days after full bloom (DAFB), and ABA treatment increased the number of lignified layers through the observation of paraffin sections (Figure 1D). Furthermore, the ‘Dangshanjinsu’ fruit surface is completely covered with rusts, accompanied by a decrease in epidermal wax compared with ‘Dangshansuli’ at 175 DAFB. Application of ABA increased the suberin layer of the ‘Dangshanjinsu’ epidermis. These results indicate the crucial role of ABA in the formation of russet pear skin.

In addition, to analyze the effect of ABA treatment on pear exocarp components, we measured each component of pear exocarp by GC–MS (Table 1). Suberin monomers, including octanedioic acid, eicosanebioic acid, docosanedioic acid, and dimethyl ester, were specific to ‘Dangshanjinsu’, and were upregulated after ABA treatment. The contents of nonanedioic acid, eicosanoic acid, cinnamic acid, hexadecanedioic acid, and linolenic acid in ‘Dangshanjinsu’ were higher than those in ‘Dangshansuli’, and they were all upregulated after ABA treatment. Conversely, the contents of m-anisic acid, hexadecanoic acid, methyl ester, and 9-octadecenoic acid were higher in green skin ‘Dangshansuli’ but there was no significant difference after ABA treatment. These results indicate the positive regulation of ABA in russet pear skin formation.

**Table 1:** Chemical compound composition of the pear exocarp.

**Description**	**Molecular formula**	**Chemical compound content (μg/mg)**		
		**WT**	**MT**	**MT-ABA**
Octanedioic acid	C_10_H_18_O_4_	0	0.09*b*	0.23*a*
Docosanedioic acid, dimethyl ester	C_24_H_46_O_4_	0	3.04*b*	4.80*a*
Eicosanebioic acid	C_22_H_42_O_4_	0	1.46*b*	2.29*a*
Linolenic acid	C_21_H_38_O_2_	6.89*b*	7.17*b*	8.94*a*
Octadecanoic acid	C_21_H_44_O_2_	0.45*b*	0.43*b*	0.66*a*
Eicosanoic acid	C_21_H_42_O_2_	0.64*c*	1.07*b*	1.60*a*
Nonanedioic acid	C_11_H_20_O_4_	0.37*c*	0.83*b*	1.40*a*
Benzoic acid	C_14_H_24_O_4_	0.65*b*	0.61*b*	0.76*a*
m-Anisic acid	C_12_H_18_O_4_	2.02*a*	0.67*b*	0.64*b*
Methyl stearate	C_19_H_38_O_2_	1.24*a*	1.01*b*	0.97*b*
Hexadecanoic acid, trimethylsilyl ester	C_19_H_40_O_2_	1.49*a*	1.18*b*	1.62*a*
Hexadecanoic acid, methyl ester	C_17_H_34_O_2_	3.20*a*	1.80*b*	2.03*b*
9-Octadecenoic acid	C_19_H_36_O_2_	9.68*a*	1.82*b*	1.41*b*
Hexadecanedioic acid	C_18_H_34_O_4_	5.83*c*	17.95*b*	26.72*a*
Cinnamic acid	C_14_H_20_O_4_	0.73*c*	1.97*b*	3.27*a*

*Lowercase letters indicate significant differences by two-tailed Student's t-test (P < 0.05)*.

### Transcriptome Profiles of Green and Russet Pear Exocarp

**Figure 2:**
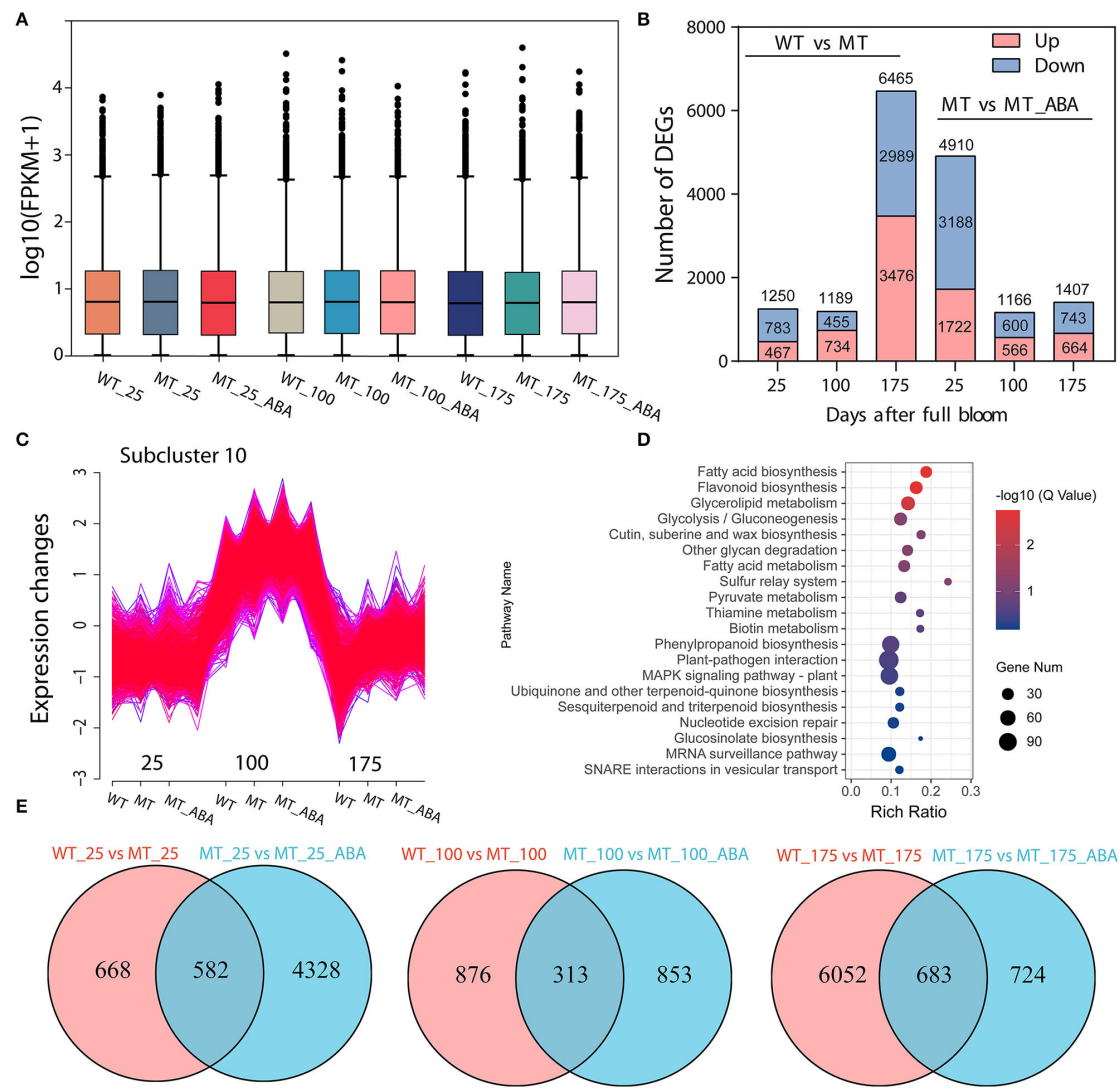
Transcriptome profiles of green (WT) and russet (MT) pear fruit skin. **(A)** Density distribution profiles of FPKM value to reflect the gene expression pattern of each sample. **(B)** Column chart of the number of DEGs. red represents upregulated DEGs, and blue represents downregulated DEGs. **(C)** Cluster analysis of the expression pattern of genes. **(D)** KEGG enrichment of genes in subcluster 10. **(E)** Venn diagram of DEGs.

RNA sequencing (RNA-Seq) was conducted using skin tissues of ‘Dangsahnsuli’ (WT), ‘Dangshanjinsu’ (MT) and ‘Dangshanjinsu’ treated with ABA (MT_ABA) at 25, 100, and 175 DAFB, respectively. A total of 34,682 expressed genes were identified, including 2,261 new genes. Reads successfully mapped to the reference genome ranged between 71.21% and 75.48%, 73.87% on average (Supplementary Table S1). Density distribution profiles of FPKM were established to reflect the gene expression pattern of each sample (Figure 2A). Transcript analysis of the two comparison groups by RNA-Seq identified 1,250, 1,189, and 6,735 DEGs at 25, 100, and 175 DAFB between WT and MT, respectively, and 4,910, 1,166, and 1,407 between MT and MT_ABA, respectively (Figure 2B). In addition, we conducted time series analysis and grouped all of the DEGs into 10 clusters (Supplementary Figure S1). We observed that the genes in cluster 10 were highly expressed at 100 DAFB and showed increased expression after ABA treatment (Figure 2C). KEGG enrichment analysis of 2,870 genes in subcluster 10 showed that fatty acid biosynthesis, cutin, suberin, and wax biosynthesis (CSW), and PPP were significantly enriched (Figure 2D). This evidence suggests that ABA treatment has an effect on the pathway related to russet fruit skin formation.

In addition, a Venn diagram revealed that 582, 313, and 683 DEGs were upregulated or downregulated uniquely in the WT-MT and MT-MT_ABA comparison groups, respectively (Figure 2E). Furthermore, KEGG enrichment analysis of DEGs in the three developmental stages showed that PPP, CSW, and α-linolenic acid metabolism were significantly enriched at 100 DAFB and 175 DAFB, respectively (Supplementary Figure S2). Interestingly, these pathways were also significantly enriched between MT and MT_ABA. Overall, ABA promotes russet pear skin formation by regulating the expression of genes involved in these pathways.

### DEGs Involved in Russet Pear Skin Formation

DEGs in pathways involved in suberin biosynthesis were investigated and showed that the expression of genes involved in fatty acid elongation had downregulated expression, including *KCS11, KCS4, KCS9, KCS19, KCS10*, and *KCS6* (Supplementary Figure S3). Genes involved in the CSW pathway, including *CYP704C1, FAR3*, and *CYP94A2* were upregulated, while *CER1* and *CYP86A22* were downregulated (Supplementary Figure S4). The expression of key enzymes involved in lignin biosynthesis was upregulated to varying degrees during three developmental periods, including 4CL, CAD, and POD (Supplementary Figure S5). In addition, G subfamily proteins of the ABC transporter superfamily were involved in the transport of suberin (Soler et al., 2007; Landgraf et al., 2014; Hou et al., 2018) and were significantly upregulated after ABA treatment, including *ABCG5, ABCG11*, and *ABCG20* (Supplementary Figure S6). These results indicated that ABA promotes the accumulation of suberin and lignin by regulating the expression of genes at the transcript level.

### Metabolomics Analysis of Pear Exocarp

**Figure 3:**
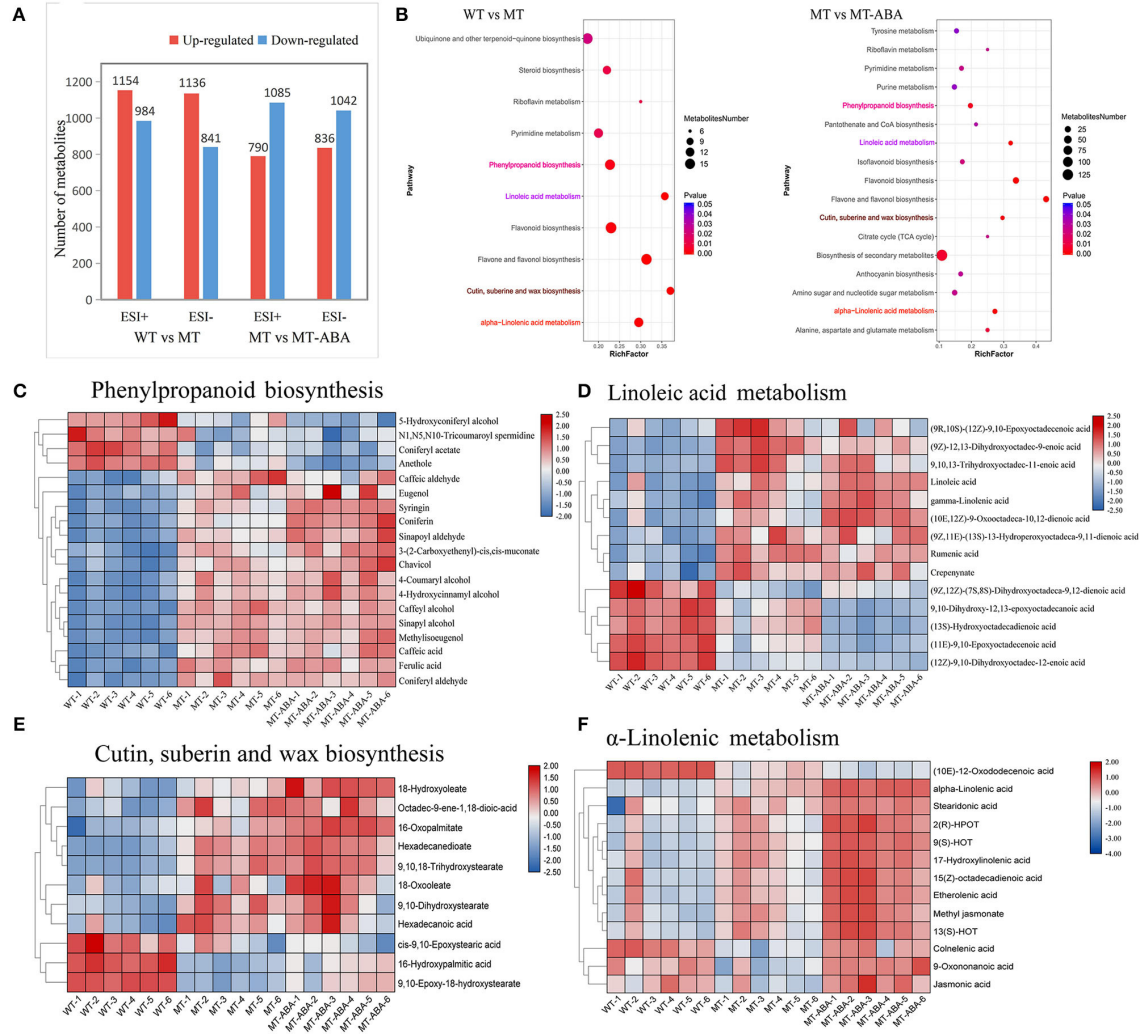
Differential metabolite analysis of pear exocarp. **(A)** Quantity statistics of DMs. **(B)** KEGG enrichment analysis of significant DMs. **(C)** Heatmap of DMs involved in phenylpropanoid biosynthesis. **(D)** Heatmap of DMs involved in linoleic acid metabolism. **(E)** Heatmap of DMs involved in cutin suberin and wax biosynthesis. **(F)** Heatmap of DMs involved in α-linolenic acid metabolism. WT, ‘Dangshansuli’. MT, ‘Dangshanjinsu’. MT-ABA, ‘Dangshanjinsu’ treated with exogenous ABA.

We characterized the exocarp of WT, MT, and ABA-treated MT metabolomic changes. A total of 10,236 and 10,148 ions were detected in positive and negative ion modes, respectively. Among them, we detected 2,138 and 1,977 differential metabolites (DMs) in WT-MT, including 1,154 and 1,136 upregulated DMs and 984 and 841 downregulated DMs in positive and negative ion modes, respectively. In addition, we detected 1,875 and 1,878 DMs, including 790 and 836 upregulated and 1,085 and 1,042 downregulated DMs, in positive and negative ion modes after ABA treatment (Figure 3A). Additionally, in the WT-MT comparison group, 2,050 DMs were annotated into 94 KEGG pathways in positive ion mode, and 1,653 DMs were annotated into 101 KEGG pathways in negative ion mode. In the MT-MT_ABA comparison group, 1,929 and 1,527 DMs were categorized into 97 and 92 KEGG pathways in positive and negative ion modes, respectively (Supplementary Table S2).

In addition, the KEGG enrichment analysis of DMs (removing the duplicated ions in positive and negative ion modes) showed that α-linolenic acid metabolism, cutin suberin, and wax biosynthesis, flavonoid biosynthesis, linoleic acid metabolism, and PPP were significantly enriched (Figure 3B). These pathways were also significantly enriched after ABA treatment. Then, metabolomics analysis showed that the different metabolite contents of these significantly enriched pathways. The different metabolite contents of these pathways were significantly higher in MT, suggesting that these pathways were the crucial pathways for the formation of russet pear skin (Figures 3C–F). Interestingly, ABA treatment significantly increased the DMs of suberin-related pathways, especially α-linolenic acid metabolism (Figure 3F). Furthermore, some DMs with high content in green skin were downregulated after ABA treatment. These results suggested that ABA could promote the formation of suberin in russet fruit skin.

**Figure 4:**
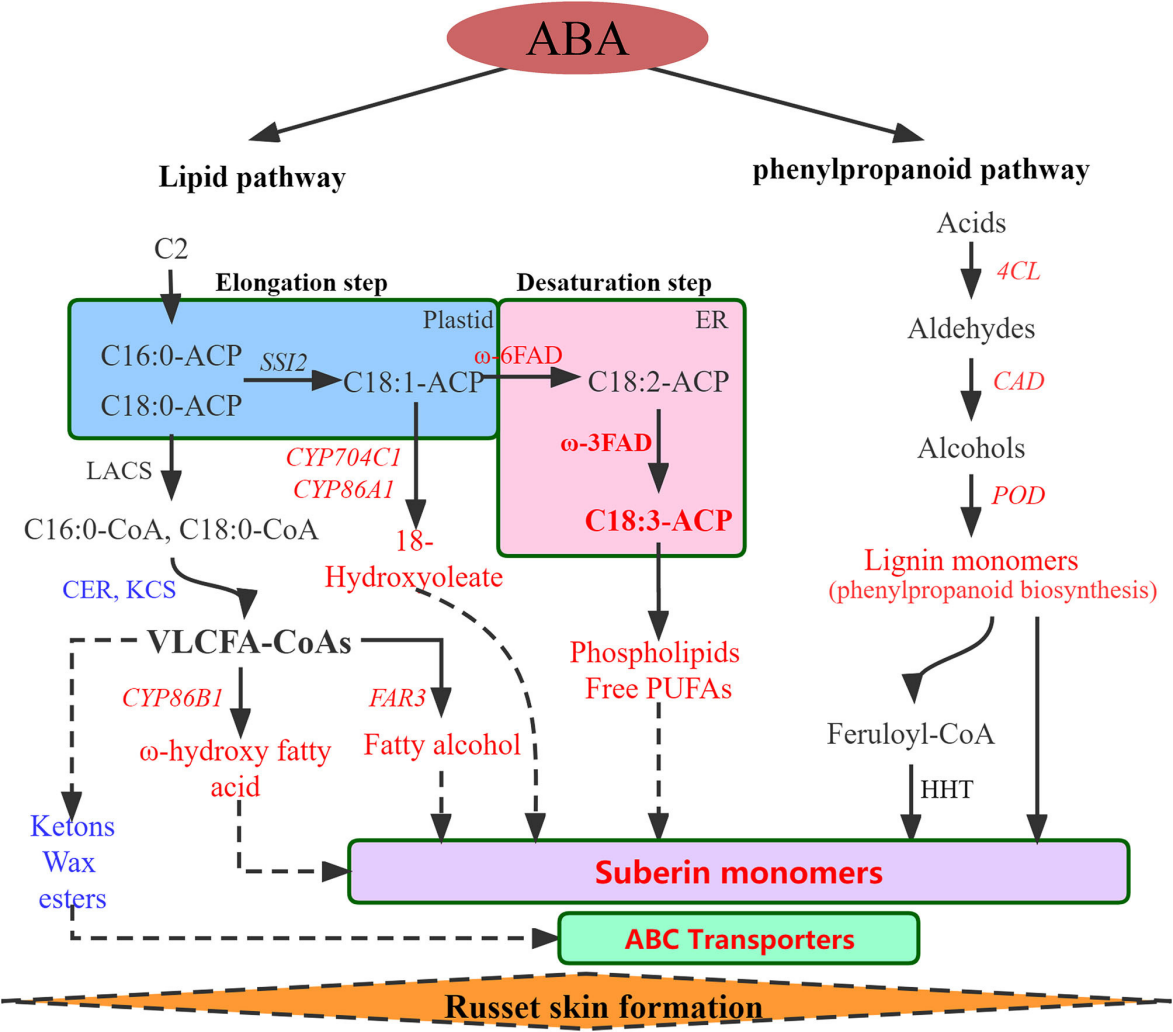
Regulatory network of the biological pathways promoted by ABA in russet fruit skin formation based on RNA-Seq and metabolomics analysis. Red represents up-regulated and blue represents down-regulated. *LACS, Long-chain acyl-CoA synthetase; CER, eceriferum; KCS, 3-ketoacyl-CoA synthase; CYP86A1, cytochrome P450 86A1; CYP86B1, cytochrome P450 86B1; CYP704C1, cytochrome P450 704C1; FAR3, Fatty acyl-CoA reductase 3; FAD, fatty acid desaturase; PUFAs, polyunsaturated fatty acid; 4CL, 4-coumarate–CoA ligase; CAD, cinnamyl alcohol dehydrogenase; POD, peroxidase; HHT. omega-hydroxypalmitate O-feruloyl transferase-like*.

In summary, a regulatory network of the biological pathways promoted by ABA in russet fruit skin formation was obtained based on RNA-seq and metabolomics analysis (Figure 4). The accumulation of suberin and lignin and the inhibition of wax induced by the application of ABA promoted the russet pear skin formation. According to the above results, we hypothesized that the increase in α-linolenic acid content induced by ABA treatment resulted in the accumulation of suberin substances.

### Identification, Phylogenetic and Expression Analysis of Fatty Acid Desaturase Gene Family Members in White Pear

**Figure 5:**
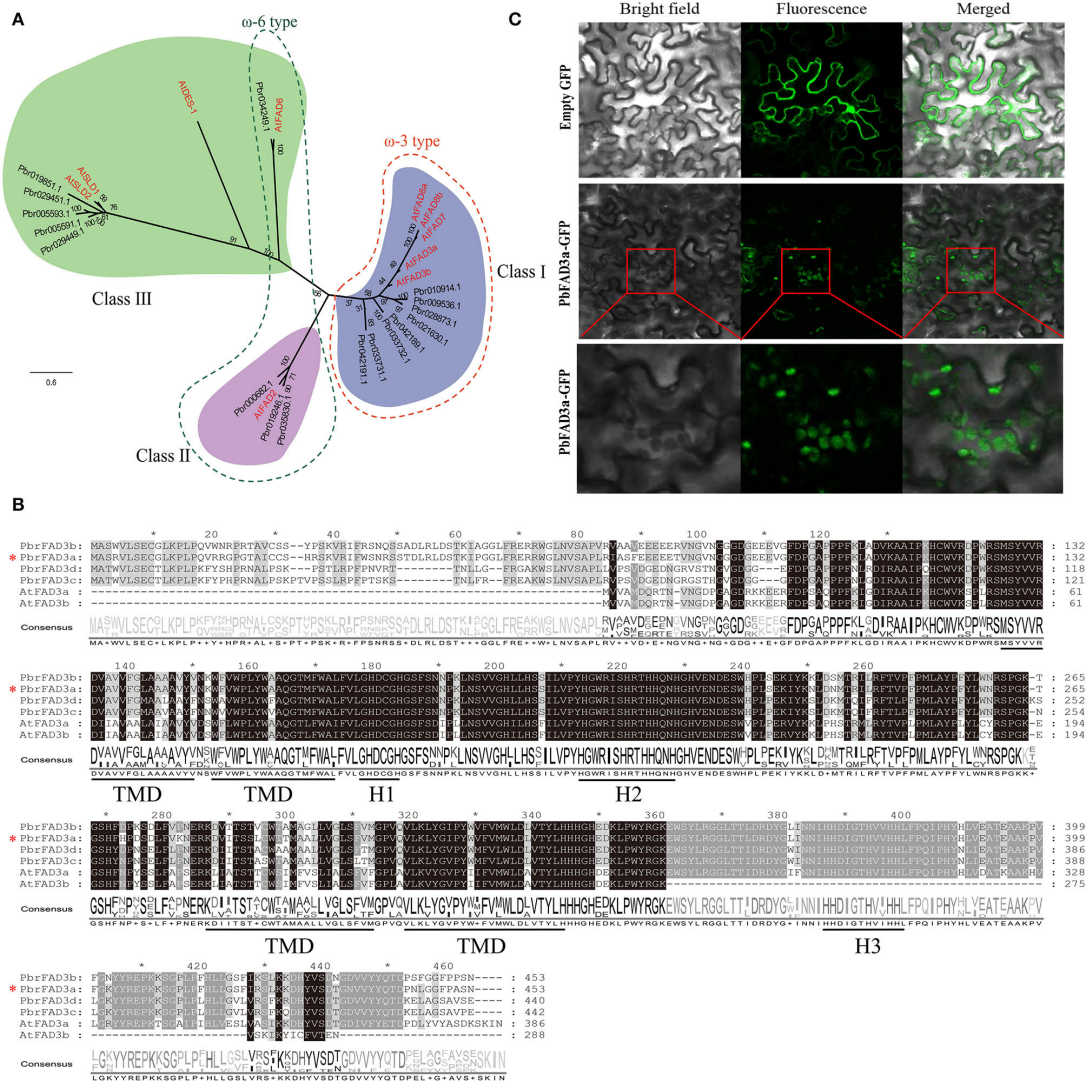
Phylogenetic, expression analysis, and subcellular localization of PbFADs. **(A)** Phylogenetic analysis of PbFADs in white pear and Arabidopsis. Alignments were calculated with Clustal X, and the phylogenetic tree was constructed using Figtree v1.4.3 software with the maximum likelihood method (bootstraps = 1,000). The position of *PbFAD3a* (*Pbr021630.1*) is indicated by an asterisk. **(B)** Sequence alignment of ω-3 FADs in pear and Arabidopsis. The sequence was aligned using Clustal X and displayed by Jalview software. Identical residues are shown on a background of black and gray. The three conserved histidine clusters (H1-H3) and the four transmembrane domains (TMD) are underlined. **(C)** Subcellular localization of *PbFAD3a* in tobacco leaves.

Thus, we performed genome-wide identification analysis to identify FAD family members in the *P. bretschneideri* genome. A total of 17 *FAD*s were identified in pear by Pfam and confirmed by inter-ProScan and several manual checks (Supplementary Table S3). A phylogenetic tree was built to show the relationship of *FAD* genes in pear and Arabidopsis (Figure 5A). Eight ω-3 FADs and four ω-6 FADs were identified. The chromosome location, gene length, and isoelectric point (PI) of the PbFADs are provided in Supplementary Table S3. Fifteen FAD genes were mapped onto chromosomes 1, 2, 3, 4, 7, 11, 12, and 15, and the other two were located on scaffold contigs. Sequence analysis showed that ω-3 FADs contained three histidine boxes (H1-H3) and four conserved transmembrane domains (TMDs; Figure 5B). Four FAD3s (FAD3a-3d) homologous to *AtFAD3* were detected in pears, and there was a high degree of sequence similarity among them, indicating that there may be functional redundancy among the PbFAD3s. Subcellular localization showed that the *PbFAD3a* gene was localized in the chloroplastid (Figure 5C).

**Figure 6:**
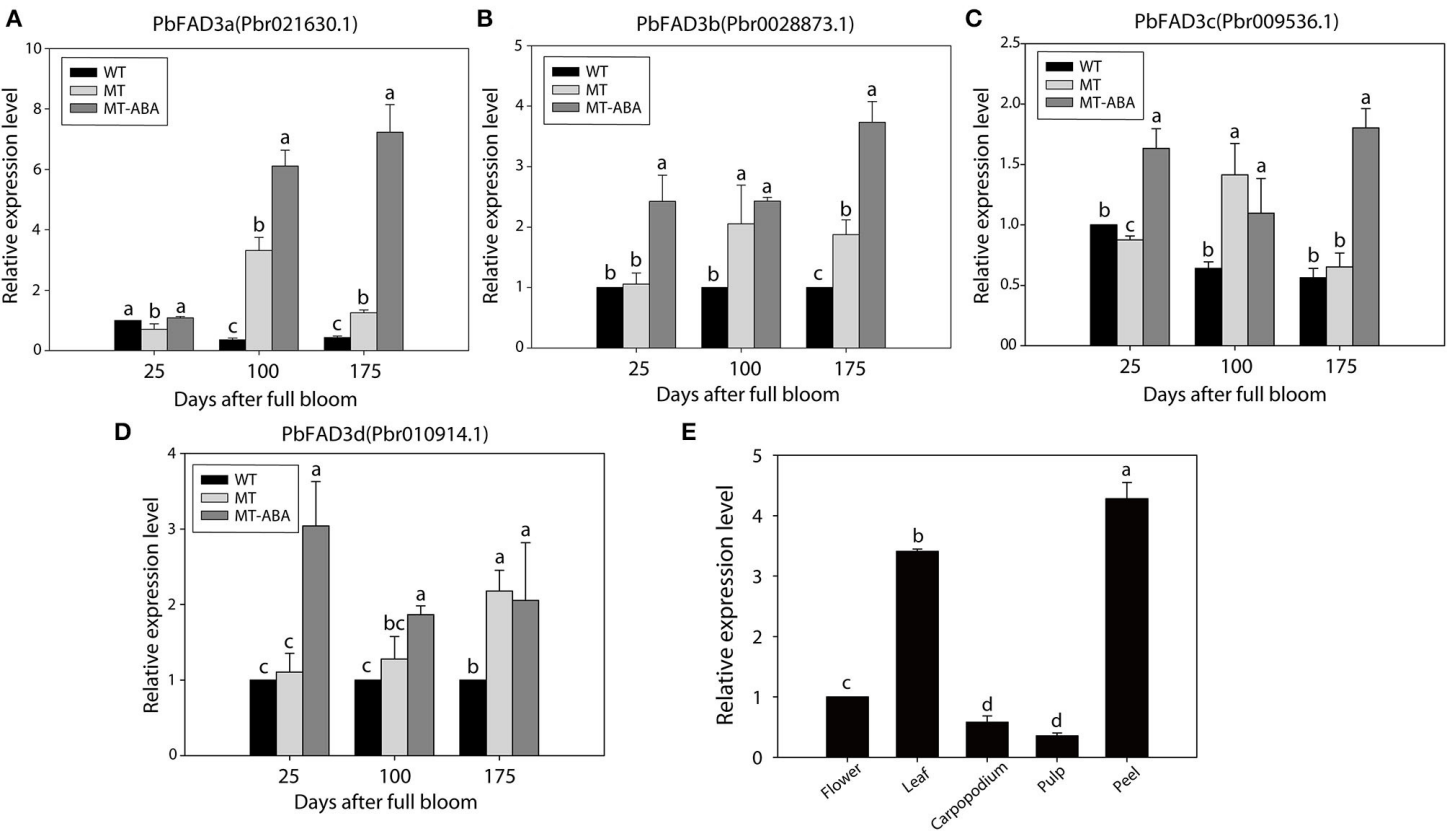
qRT-PCR verification of *PbFAD3s*. **(A–D)** Expression analysis of *PbFAD3s*. **(E)** Tissue specificity analysis of *PbFAD3a*. WT, ‘Dangshansuli’; MT, ‘Dangshanjinsu’; MT-ABA, ‘Dangshanjinsu’ treated with exogenous ABA (100 μM); DAFB, days after full bloom. The error bars are the means ± SD of three biological repeats and lowercase letters indicate significant differences by two-tailed Student's *t*-test (*p* < 0.01).

In addition, the expression of the four *PbFAD3s* was monitored by qRT–PCR (Figures 6A–D). We observed a significant difference in the expression levels of *PbFAD3a/3b* in two developmental stages between WT and MT, and their expression was upregulated after treatment with ABA. To further analyze the expression pattern of *PbFAD3a* in other tissues, we evaluated its expression in flowers, leaves, carpopodium, pulp and peel (Figure 6E). The results showed that *PbFAD3a* specifically expressed in pear fruit exocarp. These results indicate that *PbFAD3a* may play a critical role in the formation of russet pear skin.

### Overexpression of *PbFAD3a* in Arabidopsis Caused a Dwarf Phenotype

**Figure 7:**
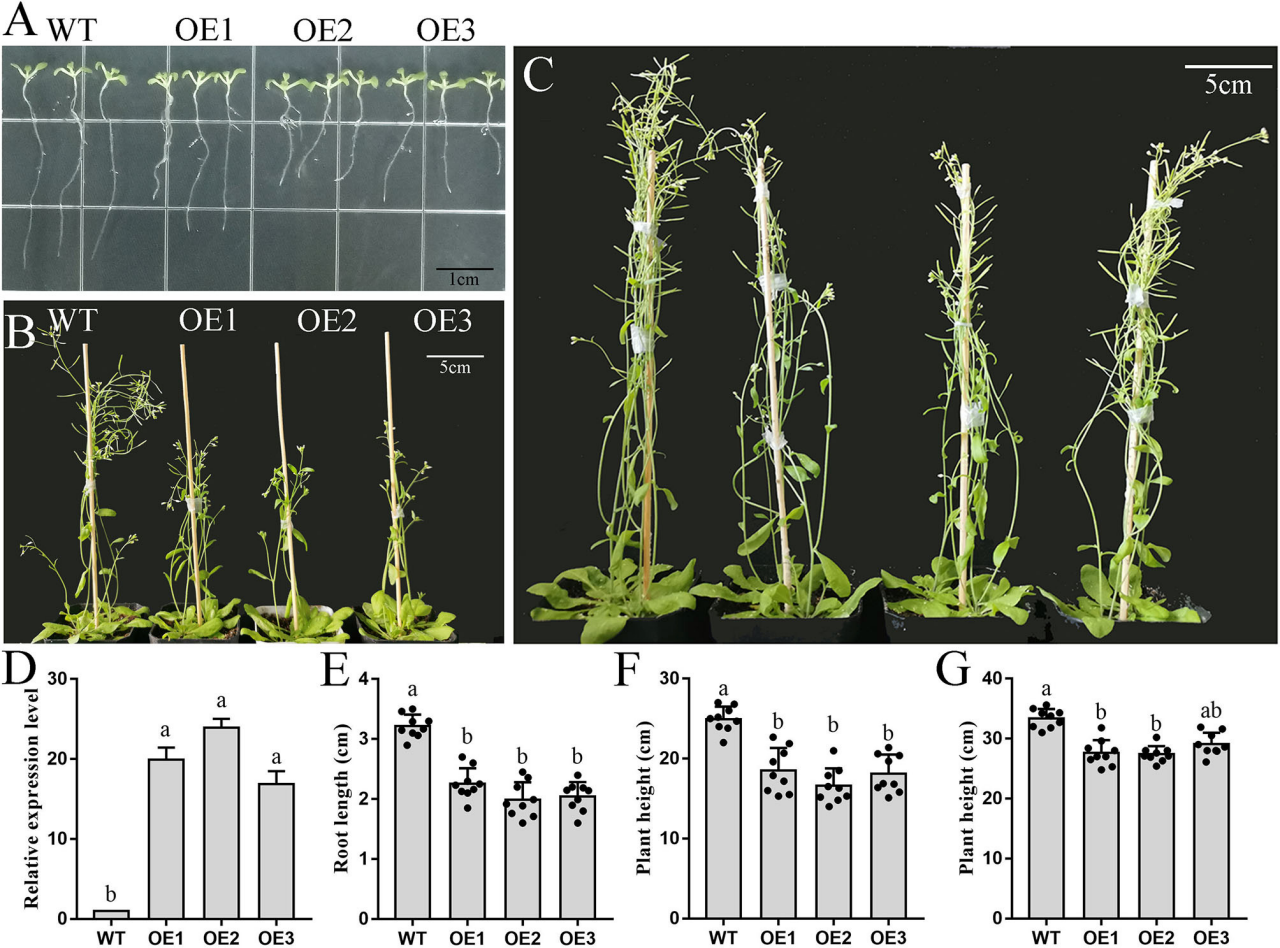
Phenotype and biomass determination of wild-type and *PbFAD3a* transgenic Arabidopsis. **(A–C)** Wild-type and transgenic Arabidopsis plants grew for 10 d **(A)**, 35 d **(B)**, and 55 d **(C)** in a long-day photoperiod. **(D)** qRT–PCR analysis showed the overexpression of *PbFAD3a* in the seedlings of representative transgenic lines. Biomass data of wild-type and *PbFAD3a* transgenic Arabidopsis plants grew for 10 d **(E)**, 35 d **(F)**, and 55 d **(G)** in a long-day photoperiod. Data are shown as mean ± SD of nine Wild-type or T3 transgenic plants (*n* = 9). Lowercase letters indicate significant differences in the two-tailed Student's *t*-test (*p* < 0.01). WT, wild-type. OE, overexpression lines.

Based on the above results, we speculated that *PbFAD3a* acts as an activator of russet pear skin formation. To verify this hypothesis, overexpression (OE) positive lines of *PbFAD3a* were obtained by Arabidopsis infection. Homozygous plants were obtained by PCR identification and screening (Supplementary Figure S7). We observed a significant increase in the number and length of the lateral roots of the transgenic lines (Figure 7A). The high expression level of *PbFAD3a* was further confirmed in T3 generation homozygote plants using qRT–PCR analysis (Figure 7D).

We used the T3 generation of three transgenic lines (OE1, OE2, and OE3) to analyze the plant phenotypes. The wild-type (WT) and *PbFAD3a*-OE1, OE2, and OE3 were planted in MS medium, and the root length of 30 seedlings was monitored after 10 days. These results showed that the root length of the OE lines was significantly shorter than that of WT (3.17 cm), with an average reduction of 1.33 cm in the three transgenic Arabidopsis strains (Figures 7A,E). Then, the OE lines and WT were grown under long-day conditions, and it was found that the transgenic lines grew significantly slower after 5 weeks (Figures 7B,F). At this time, the plant height of the WT was 25.10 cm, while that of the OE lines was 15.51 cm. After 8 weeks of growth, the plant height of the OE lines was still lower than that of WT (Figures 7C,G). Measurements of root length and plant height showed that the inflorescence stems of WT grew faster, and that of OE lines of *PbFAD3a* led to dwarfing phenotypes in transgenic Arabidopsis plants. These results demonstrate phenotypic changes in Arabidopsis after overexpression of *PbFAD3a*.

### *PbFAD3a* in Response to ABA Involved in Suberin Deposition

**Figure 8:**
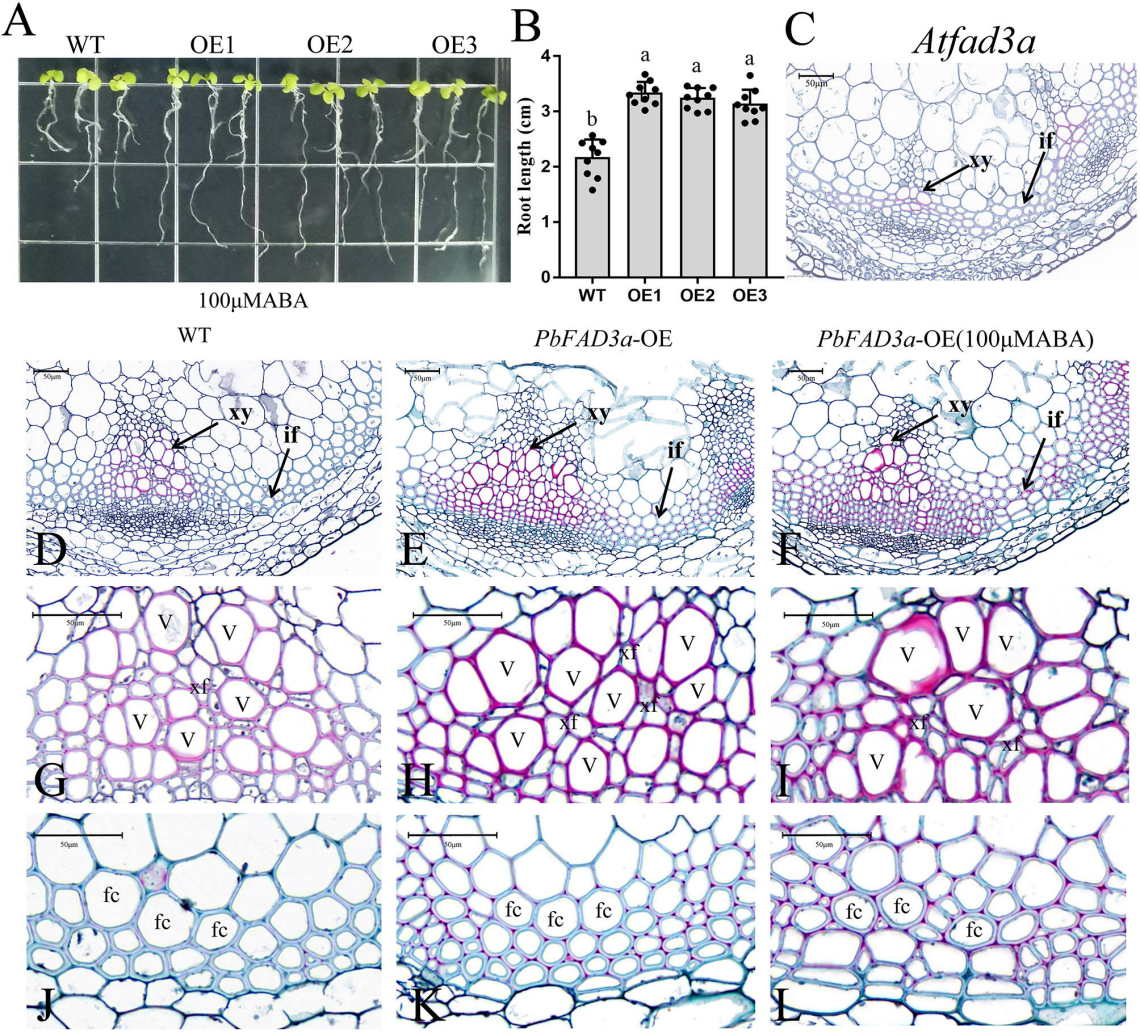
Functional analysis of *PbFAD3a* in response to ABA. **(A)** ABA treatment of transgenic Arabidopsis seedlings. **(B)** Root length of PbFAD3a-transformed OE lines in response to ABA. Cross section of Arabidopsis homologous mutant of the *PbFAD3a* **(C)**, wild-type (Col-0) **(D,G,J)**, PbFAD3a-transformed Arabidopsis **(E,H,K)**, and *PbFAD3a* transgenic line treated with 100 μM ABA **(F,I,L)**. Cross section stained with Safranin O-Fast green. The suberized, lignified, and fibrotic cell walls are stained with reddish-brown, red, and green, respectively. WT, wide-type. OE, Overexpression. If, Interfascicular fiber. Xy, Xylem. V, Vessel cell. Fc, fiber cell. Xf, Xylary fiber. The vertical bars are the means ± SD of three biological replicates and lowercase letters indicate significant differences by two-tailed Student's *t*-test (*P* < 0.01).

To verify that *PbFAD3a* is involved in the biosynthesis of suberin in response to ABA signals, WT Arabidopsis and three transgenic lines were planted in MS medium with 100 μM ABA (Figure 8A). We observed that the root length of the transgenic lines was significantly longer than that of the WT after 2 weeks of growth (Figures 8A,B). Interfascicular fibers and xylem cells are the main stem tissues supporting the upright growth of Arabidopsis inflorescences.

To further observe the changes in the interfascicular fibers and xylem vessel cells in the Arabidopsis stems, the paraffin section staining of the stems was conducted (Figures 8C–L). Cross-section staining of the Arabidopsis stems showed that the xylem and intervascular fibers of the *AtFAD3a* mutant were less stained than those of WT, indicating a decrease in suberification and lignification (Figure 8C). However, the xylem and intervascular fibers of the transgenic lines were more stained than those of the WT, suggesting that *PbFAD3a* may promote cell suberification and lignification in transgenic Arabidopsis stems (Figures 8G,H,J,K). Furthermore, we treated transgenic Arabidopsis with ABA during the growth period and found that the xylem vessel cells and fiber cells were stained deeply, indicating that ABA generated a higher degree of suberification (Figures 8I,L). In addition, we statistically analyzed the germination rate and found that the germination rate of the transgenic lines was significantly higher than that of the wild type after adding different concentrations of ABA to the substrate (Supplementary Figure S8).

**Figure 9:**
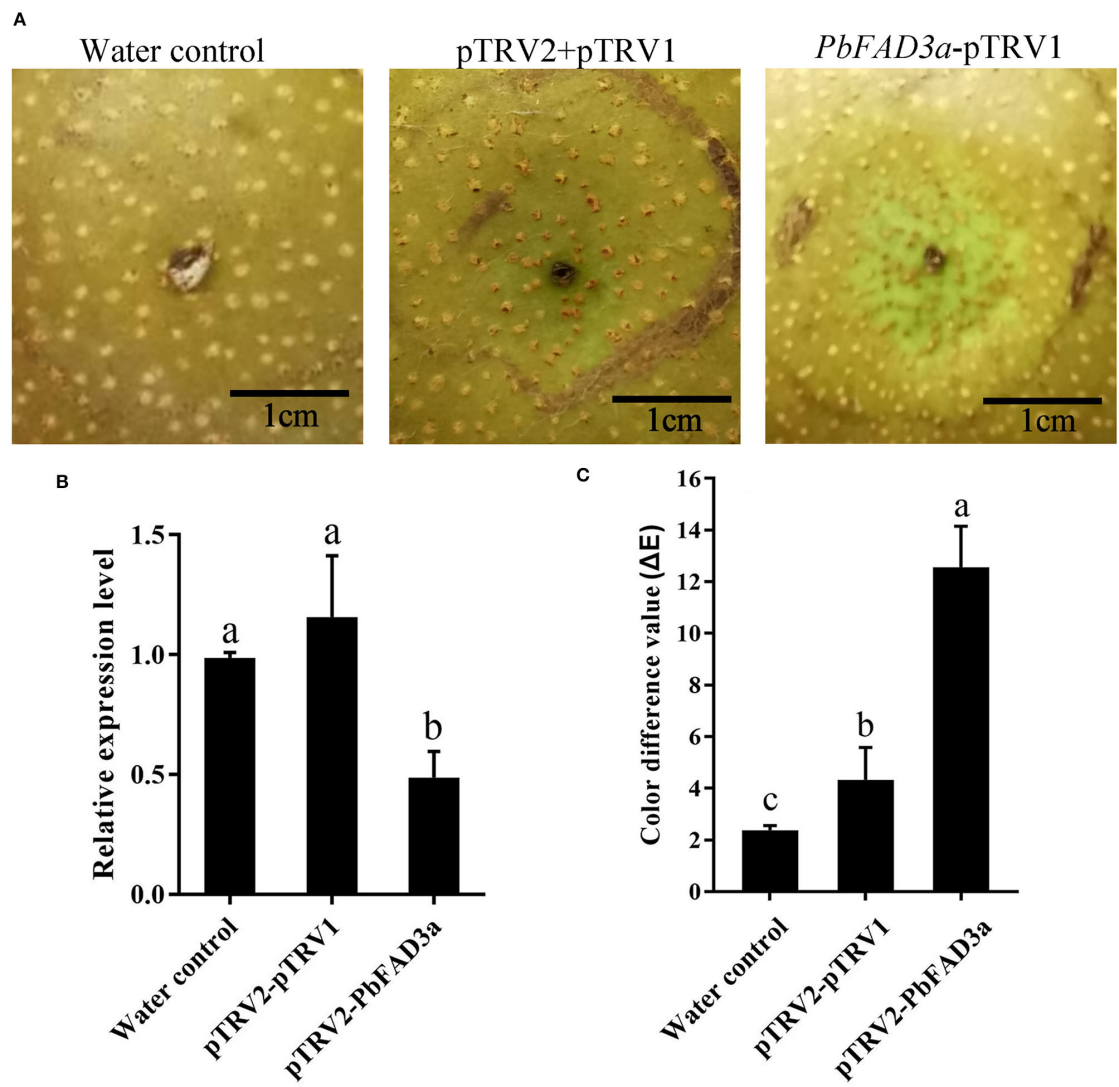
The positive regulation of *PbFAD3a* in russet pear skin formation. **(A)** Virus-induced gene silencing (VIGS) of *PbFAD3a* in ‘Xiangnan’ pear peel at 35DAFB. **(B)** Relative expression level of *PbFAD3a* after 14 days after infiltration. **(C)** Color difference of pear exocarp after silencing of *PbFAD3a*. Phenotype was observed 14 days after infiltration. The error bars are the means ± SD of three biological repeats and lowercase letters indicate significant differences by two-tailed Student's *t*-test (*p* < 0.01).

Furthermore, a VIGS assay was performed to verify the function of *PbFAD3a* in russet skin formation. The accumulation of russet peel was significantly inhibited after VIGS infiltration (Figure 9A) and the expression of *PbFAD3a* was downregulated (Figure 9B). The chromatic aberration was significantly different from the negative control and water treatment (Figure 9C). These results indicated that *PbFAD3a* plays a crucial role in russet pear skin formation.

**Figure 10:**
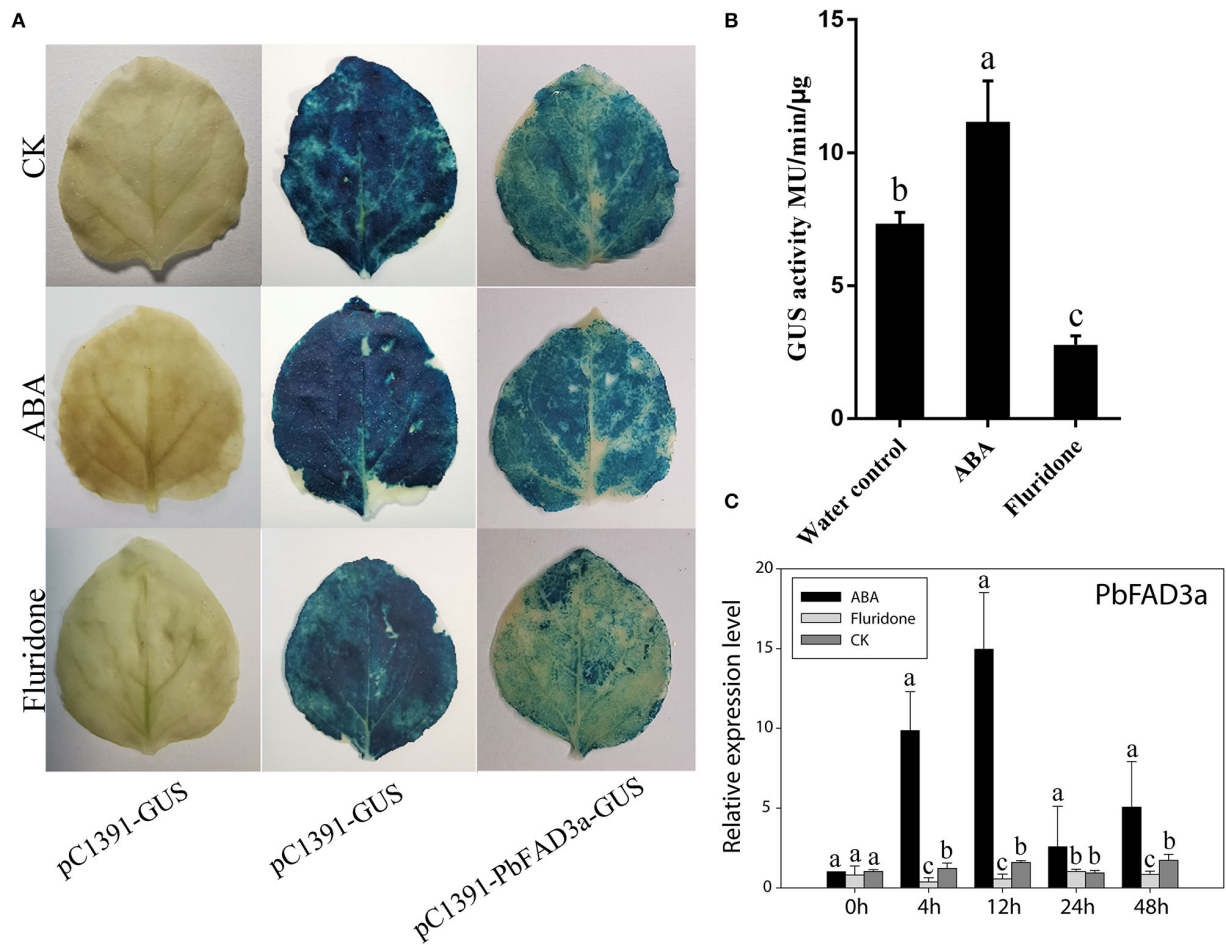
*PbFAD3a* involved in russet pear skin formation in response to ABA. GUS staining **(A)** and GUS activity **(B)** in transient *Nicotiana benthamiana* leaves after treatments of pure water, ABA and fluridone. **(C)** The expression pattern of *PbFAD3a* after ABA and fluridone treatments in pear calli. The error bars are the means ± SD of three biological repeats and lowercase letters indicate significant differences by two-tailed Student's *t*-test (*p* < 0.01).

To further confirm that the positive regulation of *PbFAD3a* induced by ABA contributes to russet skin formation, we analyzed the promoter region of pre-PbFAD3a (~ 2,000-bp upstream of *PbFAD3a*) using the PlantCARE software. There were two ABA-responsive elements in the *PbFAD3a* promoter, including ABRE and ABRE3a elements (Supplementary Table S4), suggesting a regulatory relationship between *PbFAD3a* and ABA. Histochemical analysis of GUS expression driven by the *PbFAD3a* promoter in tobacco leaves suggested that the *PbFAD3a* promoter could initiate the expression of the GUS reporter gene and ABA treatment could promote the enhancement of its activity while fluridone (an inhibitor of ABA synthesis) restrained it (Figures 10A,B). Furthermore, the expression pattern of *PbFAD3a* treated with ABA and fluridone was monitored in pear calli (Figure 10C). ABA significantly increased the expression of *PbFAD3a* after treatment for 4 and 12 h, while fluridone inhibited it. These results suggest that the positive regulation of *PbFAD3a* induced by ABA contributes to the russet skin formation in the mutant of ‘Dangshansuli’.

### *MYB1R1* and *MYC2* Are Induced by ABA and Bind to the *PbFAD3a* Promoter to Activate Its Expression

**Figure 11:**
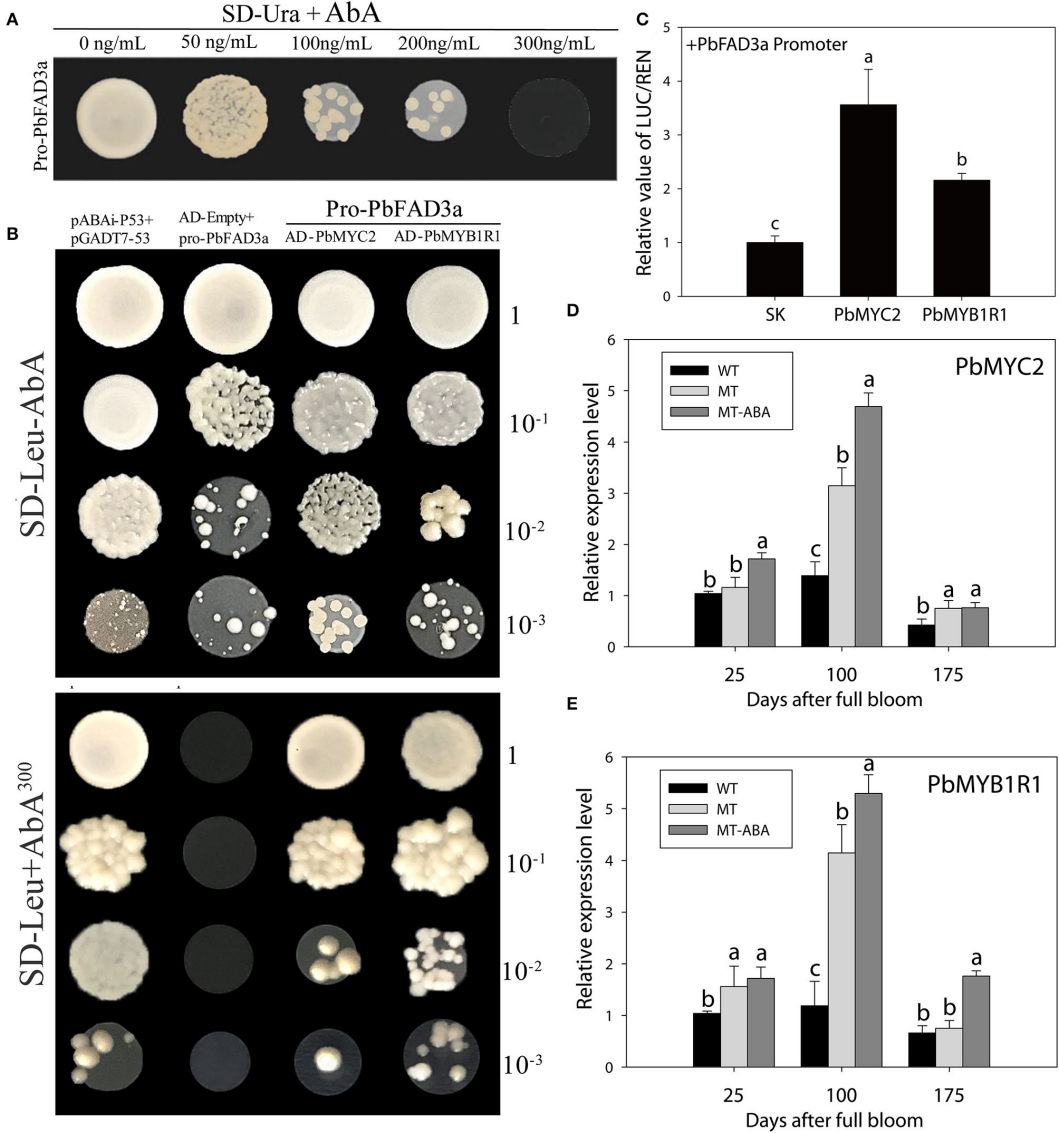
*MYB1R1* and *MYC2* are induced by ABA and bind to the *PbFAD3a* promoter to activate its expression. **(A)** Screening of ABA concentration. **(B)** Y1H assays. AD-53 was co-transformed with the pAbAi-p53 to Y1HGold as positive control, while AD-empty and pro-PbFAD3a-AbAi were used as negative controls. **(C)** Dual luciferase assays demonstrating that *MYC2* and *MYB1R1* activate the *PbFAD3a* promoter. The ratio of LUC/REN of the empty vector (SK) plus the promoter was used as the reference (set as 1). **(D)** qRT–PCR analysis of *PbMYC2* **(D)** and *PbMYB1R1* **(E)** after ABA treatment. Pro, promoter. The error bars are the means ± SD of three biological repeats and lowercase letters indicate significant differences by two-tailed Student's *t*-test (*p* < 0.01).

To examine whether the expression of *PbFAD3a* is regulated by ABA, the 1,875 bp of the *PbFAD3a* promoter was cloned and yeast one-hybrid (Y1H) screening was performed to indentify the probable factors that respond to ABA and activate *PbFAD3a* transcription. Linearized pAbAi-pro-PbFAD3a was inserted into Y1HGold and the self-activation was tested on the SD-Ura with AbA from 0 to 300 ng/mL. The results showed that the *PbFAD3a* promoter was repressed by 300 ng/mL AbA (Figure 11A). Approximately 200 positive clones were screened from the pear exocarp cDNA library and sequenced. Among the selected clones, genes that appeared at least twice were selected for further identification (Supplementary Table S6). Among them, *MYC2* and *MYB1R1* appeared five times and were selected to verifiy their interactions.

In the interaction tests, the expression of *PbMYC2* and *PbMYB1R1* separately induced the expression of the AbA-resistance reporter gene driven by the *PbFAD3a* promoter, indicating that *PbMYC2* and *PbMYB1R1* could bind to the *PbFAD3a* promoter directly (Figure 11B). Dual luciferase assays showed that *PbMYC2* and *PbMYB1R1* could significantly activate the *PbFAD3a* promoter (Figure 11C). In addition, the expression of *PbMYC2* and *PbMYB1R1* could be induced by ABA, indicating that ABA induced the expression of *PbMYC2* and *PbMYB1R1* to activate the promoter of *PbFAD3a* and promote the biosynthesis of suberin (Figures 11D,E).

## Discussion

Suberin, a fatty acid polymer linked by ester bonds, is a secondary defensive tissue that is gradually formed in the developmental process of plants against environmental stresses and/or mechanical wounds (Franke and Schreiber, 2007; Landgraf et al., 2014; Wang et al., 2016). Russeting in apples and pears is a disorder of the fruit skin that results from microscopic cracks in the cuticle and the subsequent formation of a periderm (Khanal et al., 2013). ABA is a plant hormone associated with stress resistance and it is involved in the regulation of wound healing in response to ABA stress signals (Soliday et al., 1978; Leide et al., 2012). The purpose of this study was to explore how ABA affects suberin biosynthesis and russeting in pear skin. We performed treatments with exogenous ABA and found that it increased suberin accumulation and russeting in pear skin. Transcriptomics and metabolomics were used to identify the critical genes participating in russet pear skin formation. This study provides conclusive evidence that ABA regulates suberin deposition by inducing the transcription of PbFAD3s.

ABA is a hormone associated with major plant responses to stress, including pathogens, salt, temperature, mechanical damage, and drought (Finkelstein, 2013). Our study found that the content of ABA in russet pear skin was higher than that in green skin (Figures 1A,B), and this result was opposite to the findings of a study in sand pear (Wang et al., 2020). Then, we found that the deposition of russet pear skin was induced by the application of exogenous ABA (Figures 1C,D). Similar studies have been reported in Arabidopsis (Efetova et al., 2007; Barberon et al., 2016), tomato (Leide et al., 2012; Tao et al., 2016), kiwifruit (Han et al., 2017, 2018; Wei et al., 2020a), and potato (Kumar et al., 2010; Pau et al., 2013). Exogenous ABA treatment was able to enhance drought resistance by induceing the expression of suberin-related genes in Arabidopsis roots (Efetova et al., 2007). ABA supplementation in potato tissue medium resulted in the formation of suberin (Kumar et al., 2010). The suberin feruloyl transferase FHT, which accumulates in phellogen, is induced by wounding and is regulated by ABA in potato (Pau et al., 2013). In addition, suberin increased dramatically in the wounded tomato fruit, accompanied by an increase in ABA content (Tao et al., 2016). Similarly, ABA also accelerated wound-healing in wounded kiwifruit (Han et al., 2017; Wei et al., 2020b). Consequently, this evidence indicates that the formation of russet pear skin is dependent on ABA.

Genes and pathways involved in russet skin formation were identified via RNA-Seq and metabonomics (Supplementary Figures S1–S3). The regulation of the transcription of key pathway genes induced by ABA was also studied (Supplementary Figures S4–S7). Based on the RNA-Seq database, a regulatory network of ABA promoting russet fruit skin formation was constructed (Supplementary Figure S8). In addition, DMs of suberin-related pathways were also identified via metabolomics (Figure 2). Interestingly, the identification of differential metabolites and GC–MS analysis of pear exocarp indicated that ABA treatment significantly promoted the biosynthesis of linolenic acid, and it had a higher content in the russet mutant ‘Dangshanjinsu’ than that in ‘Dangshansuli’ (Figure 2F, Table 1). α-Linolenic acid metabolism was also significantly enriched after ABA treatment in the RNA-Seq data (Supplementary Figure S3). Polyunsaturated fatty acids (PUFAs) are important components of biological membranes and play a crucial role in regulating the normal biological functions of cells (Kang, 2003). α-Linolenic acid (C18:3), a PUFA containing three double bonds, may play a critical role in the formation of russet pear skin based on metabonomics and GC–MS analysis (Figure 2F, Table 1). All of this evidence suggests that ABA may promote the formation of russet pear skin by increasing the content of α-Linolenic acid.

In *Arabidopsis thaliana*, the conversion from C18:1 to C18:2 is catalyzed by ω-6 FADs (*AtFAD2, AtFAD6*) and the conversion from C18:2 to C18:3 is catalyzed by ω-3 FADs (*AtFAD3, AtFAD7*, and *AtFAD8*). Desaturation reactions catalyzed by *FAD2* and *FAD3* have been reported to occur in the endoplasmic reticulum, while *FAD6, FAD7*, and *FAD8* are localized in chloroplasts (Wallis and Browse, 2002). In this study, 17 *FAD* genes were identified in Chinese white pear (Supplementary Table S3). Sequence analysis showed that four FAD3s (FAD3a-3d) homologous to *AtFAD3* were detected in pears. The role of *PbFAD3a* in promoting suberification was also demonstrated by overexpression in Arabidopsis and VIGS assays in fruit skin. Similarly, in *Camelina sativa*, enhancing the expression of microRNA167a decreases the α-linolenic acid content and represses the expression of suberin-and lignin-related genes (Na et al., 2019). Furthermore, we found that ABA could activate the promoter activity of *PbFAD3a* by *Agrobacterium-*mediated transient injection of tobacco leaves. Our results showed that ABA promotes russet pear skin formation by inducing the expression of *PbFAD3a*, thereby increasing the content of α-Linolenic acid. A recent study demonstrated that *PoFAD3* is induced by ABA and regulates unsaturated fatty acid biosynthesis in *Paeonia ostia* (Li et al., 2022). Then, we standing on the search for the key transcriptional hubs that regulate ABA-induced suberin biosynthesis, and Y1H screening was performed to search for the possible factors responsive to ABA that activate *PbFAD3a* transcription. *MYC2* and *MYB1R1* have been shown to bind to the *PbFAD3a* promoter and are induced by ABA (Figures 11B–E).

## Data Availability Statement

The datasets presented in this study can be found in online repositories. The names of the repository/repositories and accession number(s) can be found below: https://dataview.ncbi.nlm.nih.gov/object/PRJNA807059.

## Author Contributions

QW and WH conceived and designed the study. LW and MW conducted the induction of pear calli. YL and XW performed Y1H assays. LL and JL conducted the determination of physiological data. BJ and ZY collected samples and prepared for RNA. QW and XT contributed to the data analysis and prepared the figures and tables. QW wrote the manuscript. LZ, ST, and WH revised the manuscript. All authors read and approved the final manuscript.

## Funding

This project was supported by the National Natural Science Foundation of China (31972985), the China Agriculture Research System of MOF and MARA (CARS-29-14), and Anhui Province Fruit-Tree Industry Technology System (AHCYTX-10).

## Conflict of Interest

The authors declare that the research was conducted in the absence of any commercial or financial relationships that could be construed as a potential conflict of interest.

## Publisher's Note

All claims expressed in this article are solely those of the authors and do not necessarily represent those of their affiliated organizations, or those of the publisher, the editors and the reviewers. Any product that may be evaluated in this article, or claim that may be made by its manufacturer, is not guaranteed or endorsed by the publisher.

## References

[B1] Bai S. Tao R. Tang Y. Yin L. Ma Y. Ni J.. (2019). BBX16, a B-box protein, positively regulates light-induced anthocyanin accumulation by activating MYB10 in red pear. Plant Biotechnol. J. 17, 1985–1997. 10.1111/pbi.13114 30963689 PMC6737026

[B2] Barberon M. Vermeer J. E. De Bellis D. Wang P. Naseer S. Andersen T. G.. (2016). Adaptation of root function by nutrient-induced plasticity of endodermal differentiation. Cell. 28, 447–459. 10.1016/j.cell.2015.12.021 26777403

[B3] Beisson F. Li-Beisson Y. Pollard M. (2012). Solving the puzzles of cutin and suberin polymer biosynthesis. Curr. Opini. Plant Biol.15, 329–337. 10.1016/j.pbi.2012.03.003 22465132

[B4] Bernards M. A. (2002). Demystifying suberin. Can. J. Botany. 80, 227–240. 10.1139/b02-017

[B5] Charoenchongsuk N. Matsumoto D. Itai A. Murayama H. (2018). Ripening characteristics and pigment changes in russeted pear fruit in response to ethylene and 1-MCP. Horticulturae. 4, 22. 10.3390/horticulturae4030022

[B6] Dastmalchi K. Kallash L. Wang I. Phan V. C. Huang W. Serra O.. (2015). Defensive armor of potato tubers: nonpolar metabolite profiling, antioxidant assessment, and solid-state NMR compositional analysis of suberin-enriched wound-healing tissues. J. Agri. Food Chem. 5, 6810–6822. 10.1021/acs.jafc.5b03206 26166447 PMC4857770

[B7] Dewey C. N. Li B. (2011). RSEM: accurate transcript quantification from RNA-Seq data with or without a reference genome. BMC Bioinformat.12, 323–323. 10.1186/1471-2105-12-323 21816040 PMC3163565

[B8] Dunn W. B. Broadhurst D. Begley P. Zelena E. Francis-McIntyre S. Anderson N.. (2011). Procedures for large-scale metabolic profiling of serum and plasma using gas chromatography and liquid chromatography coupled to mass spectrometry. Nat. Protoc. Jun 30, 1060–1083. 10.1038/nprot.2011.335 21720319

[B9] Efetova M. Zeier J. Riederer M. Lee C. W. Stingl N. Mueller M.. (2007). A central role of abscisic acid in drought stress protection of *Agrobacterium*-induced tumors on Arabidopsis. Plant Physiology. 145, 853–862. 10.1104/pp.107.104851 17827272 PMC2048785

[B10] Finkelstein R. (2013). Abscisic Acid synthesis and response. Arabidopsis Book. 11, e0166. 10.1199/tab.0166 24273463 PMC3833200

[B11] Franke R. Schreiber L. (2007). Suberin–a biopolyester forming apoplastic plant interfaces. Curr. Opin. Plant Biol. 10, 252–259. 10.1016/j.pbi.2007.04.004 17434790

[B12] Graa J. Santos S. (2007). Suberin: a biopolyester of plants' skin. Macromol. Biosci. 7, 128–135. 10.1002/mabi.200600218 17295399

[B13] Han X. Lu W. Wei X. Li L. Mao L. Zhao Y. (2018). Proteomics analysis to understand the ABA stimulation of wound suberization in kiwifruit. J. Proteomics.173:42–51. 10.1016/j.jprot.2017.11.018 29191746

[B14] Han X. Mao L. Wei X. Lu W. (2017). Stimulatory involvement of abscisic acid in wound suberization of postharvest kiwifruit. Sci. Hortic. 224, 244–250. 10.1016/j.scienta.2017.06.039

[B15] Heng W. Liu L. Wang M. Jia B. Liu P. Ye Z. F.. (2014). Differentially expressed genes related to the formation of russet fruit skin in a mutant of ‘Dangshansuli’ pear (*Pyrus bretchnederi* Rehd.) determined by suppression subtractive hybridization. Euphytica. 196, 285–297. 10.1007/s10681-013-1032-x

[B16] Heng W. Wang M. Yang J. Wang Z. Jiang X. Zhu L. (2016). Relationship between H2O2 in polyamine metabolism and lignin in the exocarp of a russet mutant of ‘Dangshansuli’ Pear (Pyrus bretschneideri Rehd.). Plant Mol. Biol. Rep. 34, 1056–1063. 10.1007/s11105-016-0985-z

[B17] Hou Z. Jia B. Li F. Liu P. Liu L. Ye Z.. (2018). Characterization and expression of the ABC family (G group) in ‘Dangshansuli’ pear (Pyrus bretschneideri Rehd.) and its russet mutant. Genet. Mol. Biol. 41, 137–144. 10.1590/1678-4685-gmb-2017-0109 29658971 PMC5901498

[B18] Inoue E. Kasumi M. Sakuma F. Anzai H. Amano K. Hara H. (2006). Identification of RAPD marker linked to fruit skin color in Japanese pear (*Pyrus pyrifolia* Nakai). Sci. Hortic. 107, 254–258. 10.1016/j.scienta.2005.07.009

[B19] Jordon-Thaden I. E. Chanderbali A. S. Gitzendanner M. A. Soltis D. E. (2015). Modified CTAB and TRIzol protocols improve RNA extraction from chemically complex Embryophyta. Appl. Plant Sci. 3. 10.3732/apps.1400105 25995975 PMC4435465

[B20] Kanehisa M. Goto S. (2000). KEGG: kyoto encyclopedia of genes and genomes. Nucleic Acids Res. 1, 27–30. 10.1093/nar/28.1.27 10592173 PMC102409

[B21] Kang J. X. (2003). The importance of omega-6/omega-3 fatty acid ratio in cell function. The gene transfer of omega-3 fatty acid desaturase. World Rev. Nutr. Diet. 92, 23–36. 10.1159/000073790 14579681

[B22] Khanal B. P. Grimm E. Knoche M. (2013). Russeting in apple and pear: a plastic periderm replaces a stiff cuticle. AoB Plants. 5, pls048. 10.1093/aobpla/pls048 23350024 PMC3553398

[B23] Kim D. Langmead B. Salzberg S. L. (2015). HISAT: A fast spliced aligner with low memory requirements. Nat. Methods. 12, 357–360. 10.1038/nmeth.3317 25751142 PMC4655817

[B24] Kolattukudy P. E. (1984). Biochemistry and function of cutin and suberin. Can. J. Botany. 62, 2918–2933. 10.1139/b84-391

[B25] Kumar G. N. Lulai E. C. Suttle J. C. Knowles N. R. (2010). Age-induced loss of wound-healing ability in potato tubers is partly regulated by ABA. Planta. 232, 1433–1445. 10.1007/s00425-010-1269-8 20839005

[B26] Kwon Y. Han H.-H. Park H.-S. (2016). The characteristics of cork and hypodermis tissues and cracking in Asian pear (*Pyrus pyrifolia* cv. *Mansoo*). Scientia Horticulturae. 199, 224–228. 10.1016/j.scienta.2015.12.044

[B27] Landgraf R. Smolka U. Altmann S. Eschen-Lippold L. Senning M. Sonnewald S.. (2014). The ABC transporter ABCG1 is required for suberin formation in potato tuber periderm. Plant Cell. 26, 3403–3415. 10.1105/tpc.114.124776 25122151 PMC4371835

[B28] Langmead B. Salzberg S. L. (2012). Fast gapped-read alignment with Bowtie 2. Nat. Methods. 9, 357–359. 10.1038/nmeth.1923 22388286 PMC3322381

[B29] Legay S. Guerriero G. Andr,é C. Guignard C. Cocco E. Charton S.. (2016). MdMyb93 is a regulator of suberin deposition in russeted apple fruit skins. New Phytologist. 212, 977–991. 10.1111/nph.14170 27716944

[B30] Leide J. Hildebrandt U. Hartung W. Riederer M. Vogg G. (2012). Abscisic acid mediates the formation of a suberized stem scar tissue in tomato fruits. New Phytologist. 194, 402–415. 10.1111/j.1469-8137.2011.04047.x 22296281

[B31] Li Y. Wang X. Zhang X. Liu Z. a. Peng L.. (2022). Abscisic acid-insensitive 5-ω3 fatty acid desaturase3 module regulates unsaturated fatty acids biosynthesis in Paeonia ostii. Plant Sci. 317, 111189. 10.1016/j.plantsci.2022.111189 35193738

[B32] Likun W. Zhixing F. Xi W. Xiaowo W. (2010). DEGseq: an R package for identifying differentially expressed genes from RNA-seq data. Bioinformatics. 26, 136–138. 10.1093/bioinformatics/btp612 19855105

[B33] Livak K. J. Schmittgen T. D. (2001). Analysis of relative gene expression data using real-time quantitative PCR and the 2(-Delta Delta C(T)) method. Methods (San Diego, Calif). 25, 402–408. 10.1006/meth.2001.1262 11846609

[B34] Love M. I. Huber W. Anders S. (2014). Moderated estimation of fold change and dispersion for RNA-seq data with DESeq2. Genome Biol. 15, 550. 10.1186/s13059-014-0550-8 25516281 PMC4302049

[B35] Mizrahi B. Richmond E. A. (1970). Abscisic acid and transpiration in leaves in relation to osmotic root stress. Plant Physiol. 10.1104/pp.46.1.169 16657411 PMC396553

[B36] Na G. Mu X. Grabowski P. Schmutz J. Lu C. (2019). Enhancing microRNA167A expression in seed decreases the α-linolenic acid content and increases seed size in *Camelina sativa*. Plant J. 98, 346–358. 10.1111/tpj.14223 30604453

[B37] Pau B. Olga S. Marçal Soler M. M. Figueras M. (2013). The potato suberin feruloyl transferase FHT which accumulates in the phellogen is induced by wounding and regulated by abscisic and salicylic acids. J. Exp. Botany. 64, 3225–3236. 10.1093/jxb/ert163 23918964 PMC3733149

[B38] Petit J. Bres C. Just D. Garcia V. Mauxion J. P. Marion D.. (2014). Analyses of tomato fruit brightness mutants uncover both cutin-deficient and cutin-abundant mutants and a new hypomorphic allele of GDSL lipase. Plant Physiology. 164, 888–906. 10.1104/pp.113.232645 24357602 PMC3912114

[B39] Pollard M. Beisson F. Li Y. Ohlrogge J. B. (2008). Building lipid barriers: biosynthesis of cutin and suberin. Trends Plant Sci. 13, 236–246. 10.1016/j.tplants.2008.03.003 18440267

[B40] Pope J. D. Black M. J. Drummer O. H. Schneider H. G. (2017). Challenges for detecting valproic acid in a nontargeted urine drug screening method. Ther. Drug Monit. 39, 457–460. 10.1097/FTD.0000000000000417 28700524

[B41] Ranathunge K. Schreiber L. Franke R. (2011). Suberin research in the genomics era–new interest for an old polymer. Plant Scie. 180, 399–413. 10.1016/j.plantsci.2010.11.003 21421386

[B42] Rocha S. l. M. Goodfellow B. J. Delgadillo I Neto C. P. Gil A. M. (2001). Enzymatic isolation and structural characterisation of polymeric suberin of cork from *Quercus suber L*. Int. J. Biol. Macromol. 28, 107–119. 10.1016/S0141-8130(00)00163-X 11164227

[B43] Rom R. C. Carlson R. (1987). Rootstocks for Fruit Crops.

[B44] Savchenko T. Kolla V. A. Wang C. Q. Nasafi Z. Hicks D. R. Phadungchob B.. (2014). Functional convergence of oxylipin and abscisic acid pathways controls stomatal closure in response to drought. Plant Physiol. 164, 1151–1160. 10.1104/pp.113.234310 24429214 PMC3938610

[B45] Serra O. Hohn C. Franke R. Prat S. Figueras M. (2010). A feruloyl transferase involved in the biosynthesis of suberin and suberin-associated wax is required for maturation and sealing properties of potato periderm. Plant J. 62, 277–290. 10.1111/j.1365-313X.2010.04144.x 20088895

[B46] Shukla V. Han J. P. Cléard F. Lefebvre-Legendre L. Gully K. Flis P.. (2021). Suberin plasticity to developmental and exogenous cues is regulated by a set of MYB transcription factors. Proc. Natl. Acad. Sci. U.S.A. 28, 118. 10.1073/pnas.2101730118 34551972 PMC8488582

[B47] Soler M. Serra O. Molinas M. Huguet G. Fluch S. Figueras M. (2007). A genomic approach to suberin biosynthesis and cork differentiation. Plant Physiol. 144, 419–431. 10.1104/pp.106.094227 17351057 PMC1913797

[B48] Soliday C. L. Dean B. B. Kolattukudy P. E. (1978). Suberization: inhibition by washing and stimulation by abscisic acid in potato disks and tissue culture. Plant Physiol. 61, 170–174. 10.1104/pp.61.2.170 16660254 PMC1091826

[B49] Tao X. Mao L. Li J. Chen J. Lu W. (2016). Abscisic acid mediates wound-healing in harvested tomato fruit. Postharvest Biol. Technol. 118, 128–133. 10.1016/j.postharvbio.2016.04.002

[B50] Trapnell C. Roberts A. Goff L. Pertea G. Kim D. D. R. K. (2012). Differential gene and transcript expression analysis of RNA-seq experiments with TopHat and Cufflinks. Nat. Protoc. 7, 562–578. 10.1038/nprot.2012.016 22383036 PMC3334321

[B51] Wallis J. G. Browse J. (2002). Mutants of Arabidopsis reveal many roles for membrane lipids. Prog. Lipid Res. 41, 254–278. 10.1016/S0163-7827(01)00027-3 11814526

[B52] Wang Q. Gong X. Xie Z. Qi K. Yuan K. Jiao Y.. (2022). Cryptochrome-mediated blue-light signal contributes to lignin biosynthesis in stone cells in pear fruit. Plant Sci. 318, 111211. 10.1016/j.plantsci.2022.111211 35351300

[B53] Wang W. Tian S. Stark R. E. (2010). Isolation and identification of triglycerides and ester oligomers from partial degradation of potato suberin. J. Agri. Food Chem. 58, 1040–1045. 10.1021/jf902854y 20028122 PMC2810711

[B54] Wang Y. Dai M. Cai D. Shi Z. (2020). Proteome and transcriptome profile analysis reveals regulatory and stress-responsive networks in the russet fruit skin of sand pear. Horticult. Res. 7, 16. 10.1038/s41438-020-0242-3 32025319 PMC6994700

[B55] Wang Y. Dai M. Cai D. Zhang S. Shi Z. (2016). A review for the molecular research of russet/semi-russet of sand pear exocarp and their genetic characters. Sci. Hortic. 210, 138–142. 10.1016/j.scienta.2016.07.019

[B56] Wei X. Mao L. Lu W. Wei X. Han X. Guan W.. (2020a). Three transcription activators of ABA signaling positively regulate suberin monomer synthesis by activating cytochrome P450 CYP86A1 in kiwifruit. Front. Plant Sci. 10, 1650. 10.3389/fpls.2019.01650 31998339 PMC6967411

[B57] Wei X. Mao L. Wei X. Xia M. Xu C. (2020b). MYB41, MYB107, and MYC2 promote ABA-mediated primary fatty alcohol accumulation via activation of AchnFAR in wound suberization in kiwifruit. Horticulture Res. 7, 86. 10.1038/s41438-020-0309-1 32528698 PMC7261769

[B58] Weigel D. Glazebrook J. (2006). Transformation of agrobacterium using the freeze-thaw method. CSH Protocols. 2006, pdb.prot4666. 10.1101/pdb.prot4666 22484682

[B59] Wen B. Mei Z. Zeng C. Liu S. (2017). metaX: a flexible and comprehensive software for processing metabolomics data. BMC Bioinformatics. 21, 183. 10.1186/s12859-017-1579-y 28327092 PMC5361702

[B60] Wu J. Wang Z. Shi Z. Zhang S. Ming R. Zhu S.. (2013). The genome of the pear (*Pyrus bretschneideri* Rehd.). Genome Res. 23, 396–408. 10.1101/gr.144311.112 23149293 PMC3561880

[B61] Yap C. K. Eisenhaber B. Eisenhaber F. Wong W. C. (2016). xHMMER3x2: Utilizing HMMER3's speed and HMMER2's sensitivity and specificity in the glocal alignment mode for improved large-scale protein domain annotation. Biol. Direct. 29, 63. 10.1186/s13062-016-0163-0 27894340 PMC5126834

[B62] Zhang J. Zhang P. F. Zhang Y. F. Bian Y. H. Liu Z. Y. Zhang C.. (2021). An integrated metabolic and transcriptomic analysis reveals the mechanism through which fruit bagging alleviates exocarp semi-russeting in pear fruit. Tree Physiol. 24, 1306–1318. 10.1093/treephys/tpaa172 33367887

