# Supplementary material for: MYB1R1 and MYC2 Regulate ω-3 Fatty Acid Desaturase Involved in ABA-Mediated Suberization in the Russet Skin of a Mutant of ‘Dangshansuli’ (Pyrus bretschneideri Rehd.)
Source: Front Plant Sci. 2022 Jun 9;13:910938. doi: 10.3389/fpls.2022.910938 (PMC9225576; doi:10.3389/fpls.2022.910938)
Supplement: Supplementary file 1 [file Data_Sheet_4.PDF]

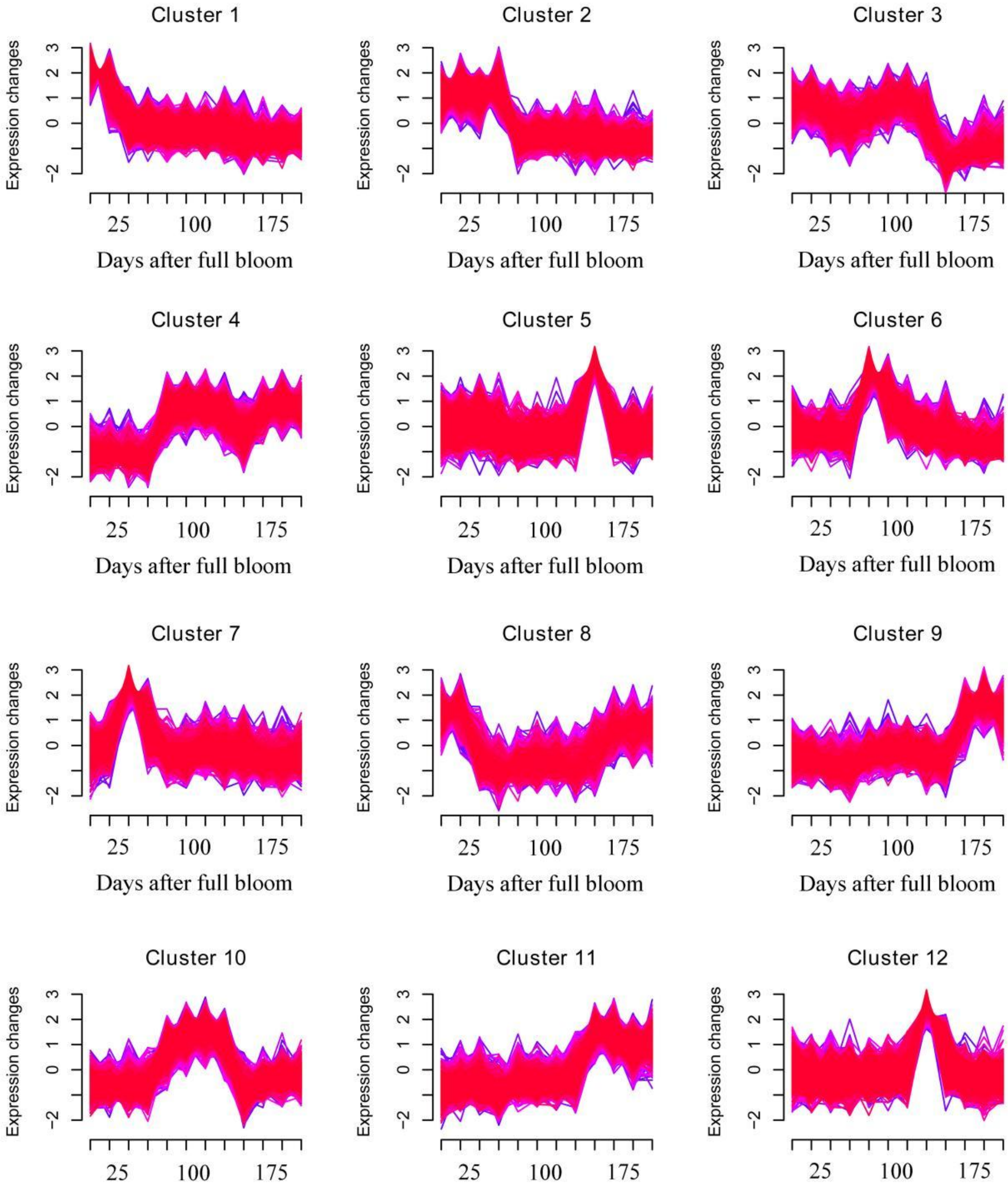

Figure S1. Time series analysis of DEGs. The diagram shows the clustering of genes into gene clusters. The vertical axis represents the trend of gene expression. Genes have similar expression patterns in samples at different time stages are grouped into the same cluster.

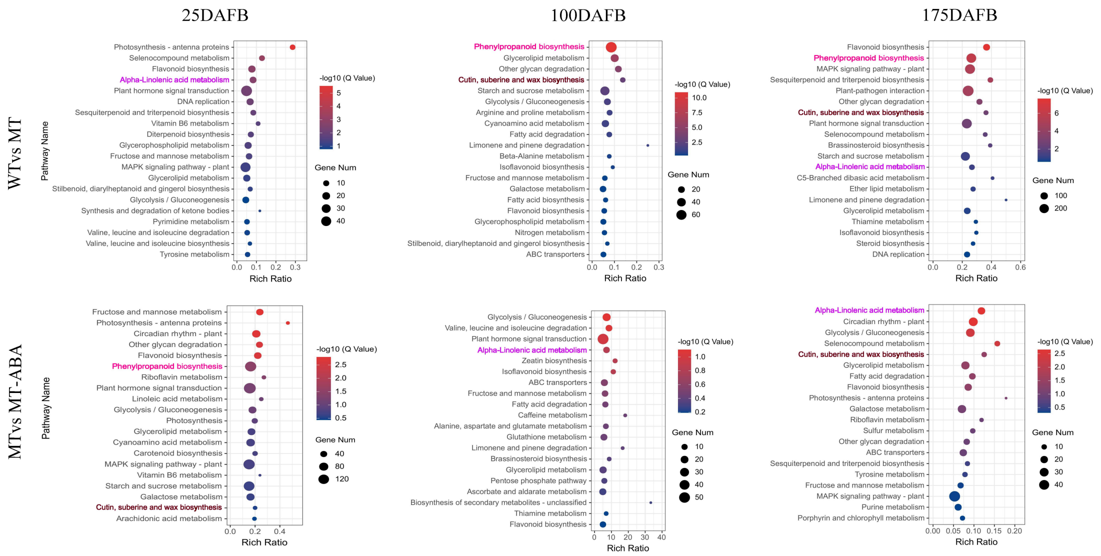

Fig. S2. KEGG enrichment bubble diagram of DEGs. A higher value indicates the higher concentration of DEGs in the pathway. A larger bubble size represents more DEGs in the pathway. The color of the bubble represents the change in the P-value determined by a hypergeometric test. A smaller P-value indicates more statistical significance and greater test reliability. DAFB, days after full bloom.

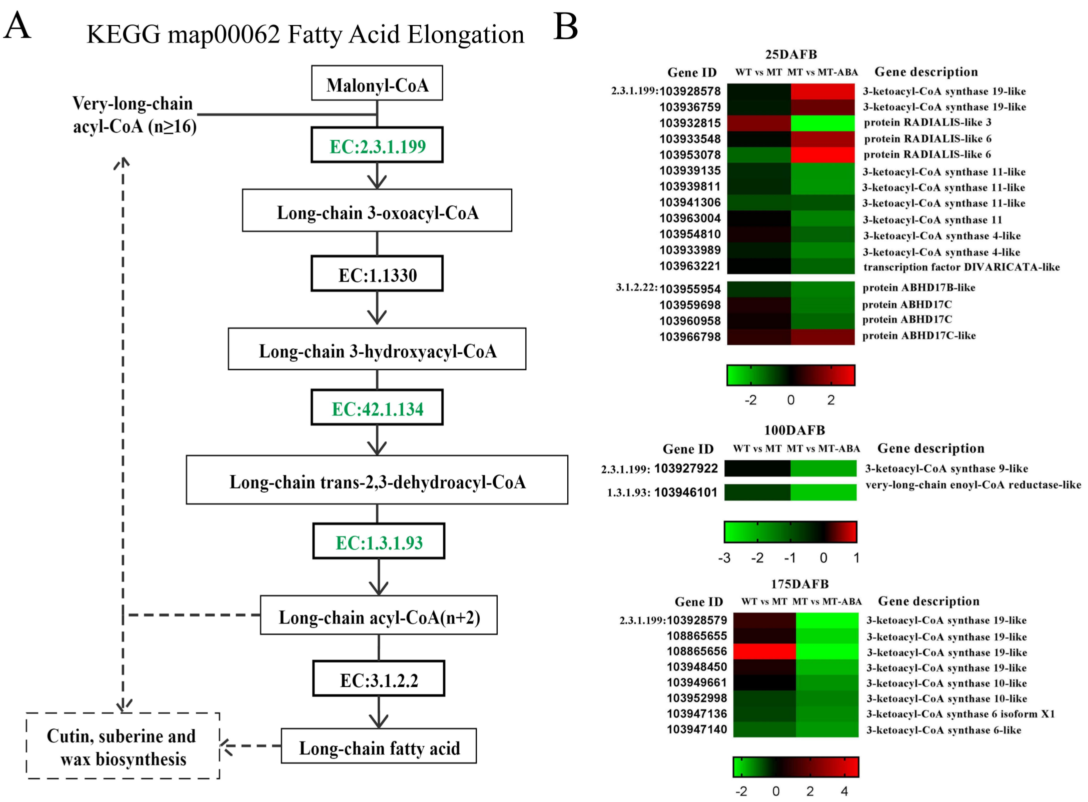

Fig. S3. Schematic diagram of ABA treatment on the regulation of fatty acid elongation pathway in pear exocarp. (A) A detailed diagram of fatty acid elongation pathway including the subset of nodes or metabolites and enzymes that are involved in the process. Red represents upregulated, while green represents downregulated. DAFB, Days after full bloom.

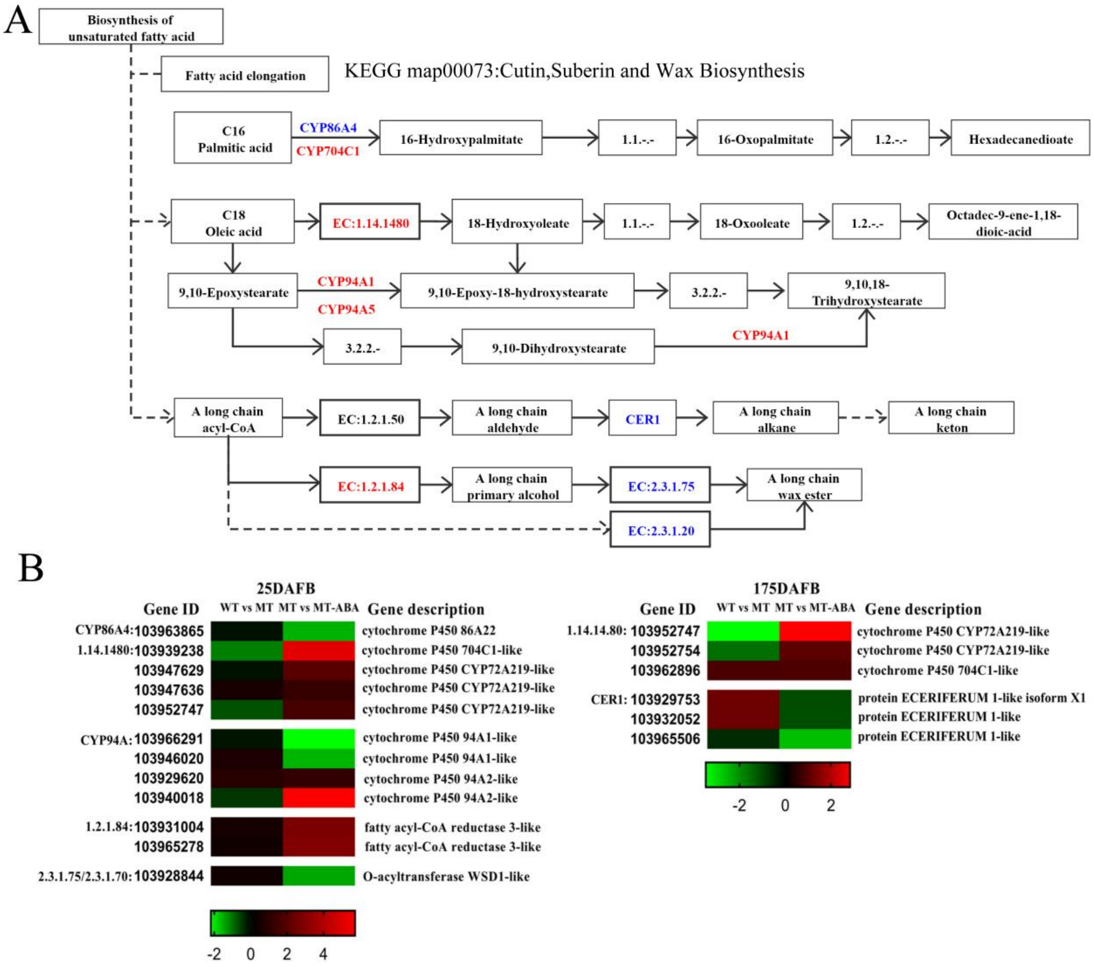

Fig. S4. Schematic diagram of ABA treatment on the regulation of cutin, suberin and wax biosynthesis in pear exocarp. (A) A detailed diagram of cutin, suberin and wax biosynthesis pathway including the subset of nodes or metabolites and enzymes that are involved in the process. Red represents upregulated, while green represents downregulated. DAFB, Days after full bloom.

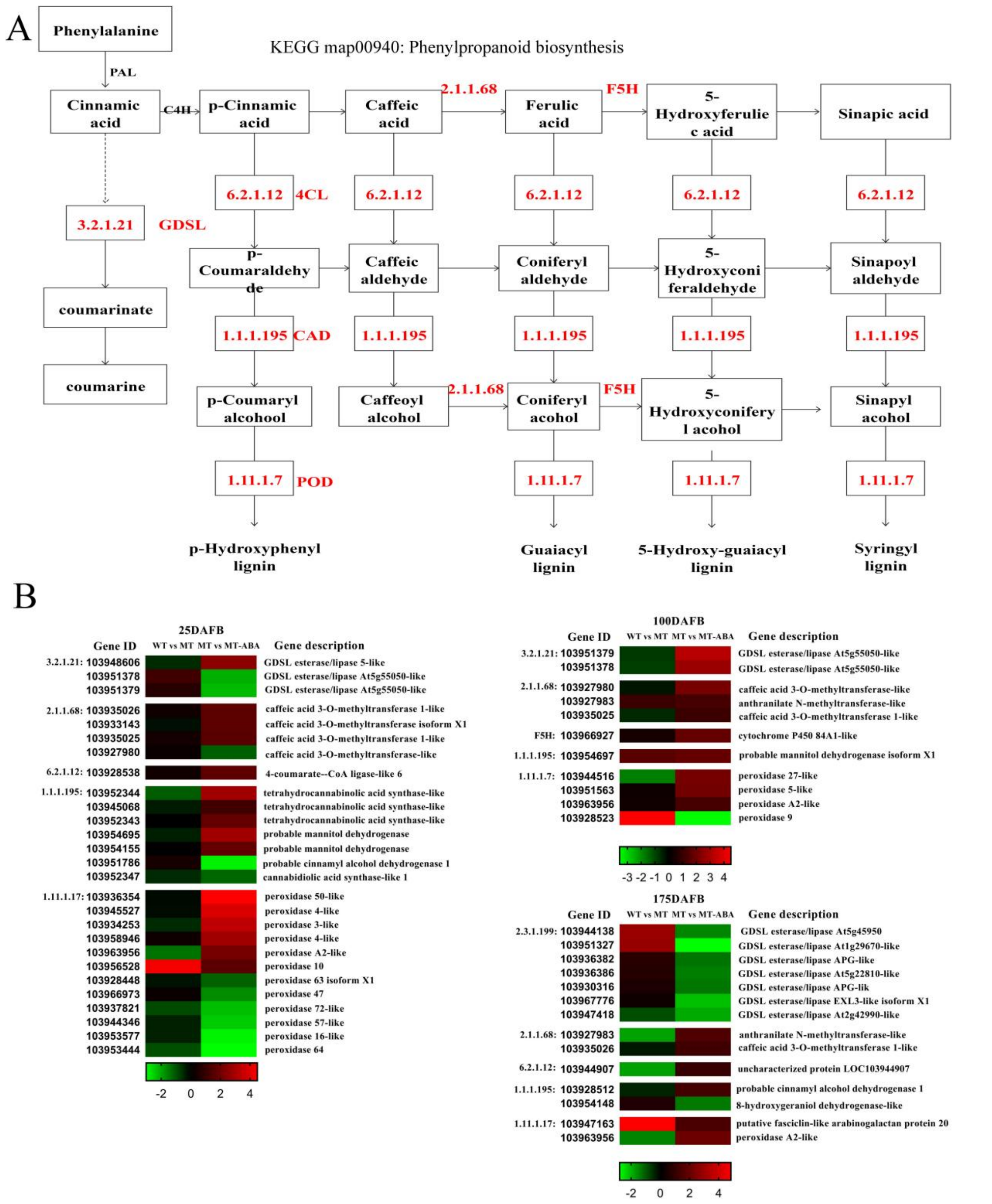

Fig. S5. Schematic diagram of ABA treatment on the regulation of phenylpropanoid biosynthesis in pear exocarp. (A) A detailed diagram of phenylpropanoid biosynthesis pathway including the subset of nodes or metabolites and enzymes that are involved in the process. Red represents upregulated, while green represents downregulated. DAFB, Days after full bloom.

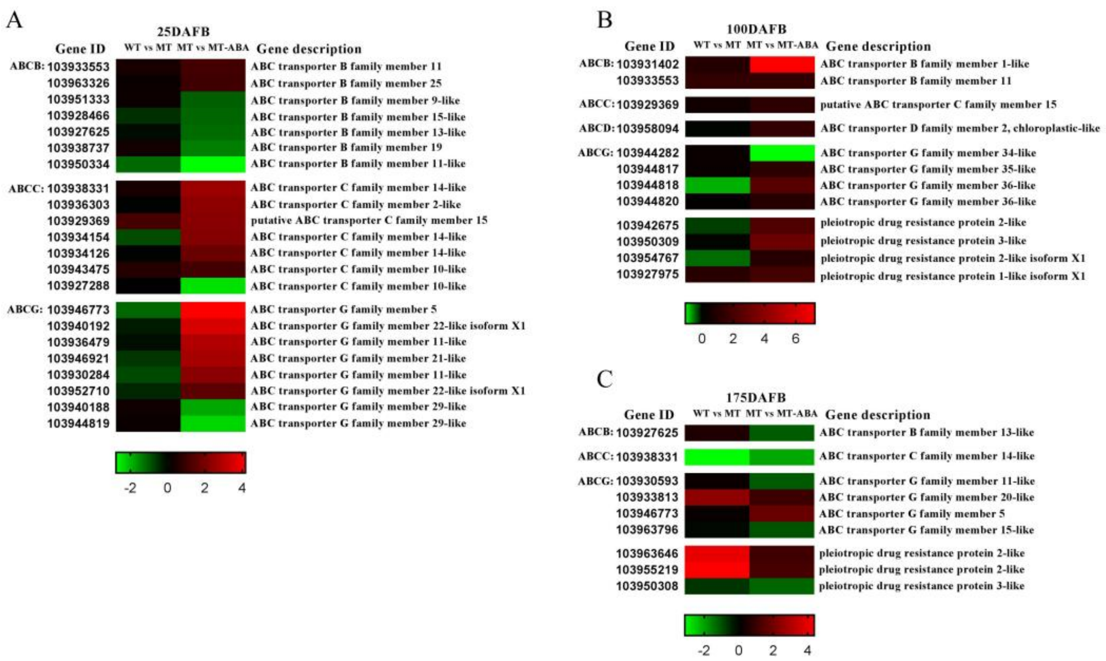

Fig. S6 Heatmap of ABA treatment on the regulation of ABC transporters in pear exocarp. Red represents upregulated, while green represents downregulated. DAFB, Days after full bloom.

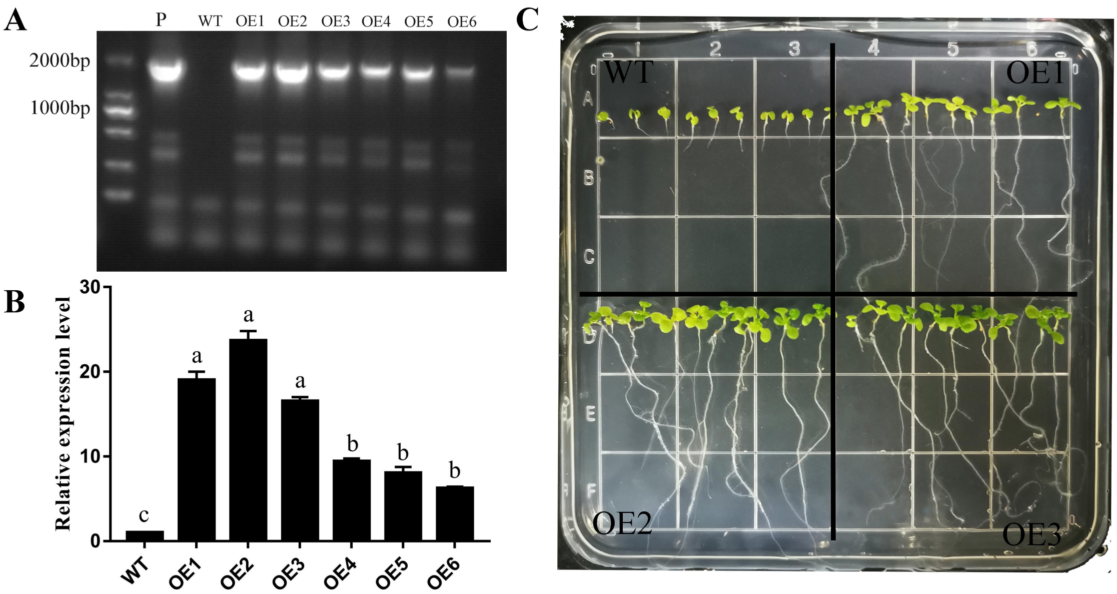

Fig. S7 Identification and screening of transgenic *Arabidopsis thaliana*. (A) PCR identification of *PbFAD3a* transgenic *Arabidopsis thaliana*. P, postive control. WT, wide type. (B) qRT-PCR monitors the expression level of *PbFAD3a* gene in transgenic lines. OE, over expression. (C) Screening of transgenic *Arabidopsis thaliana*.

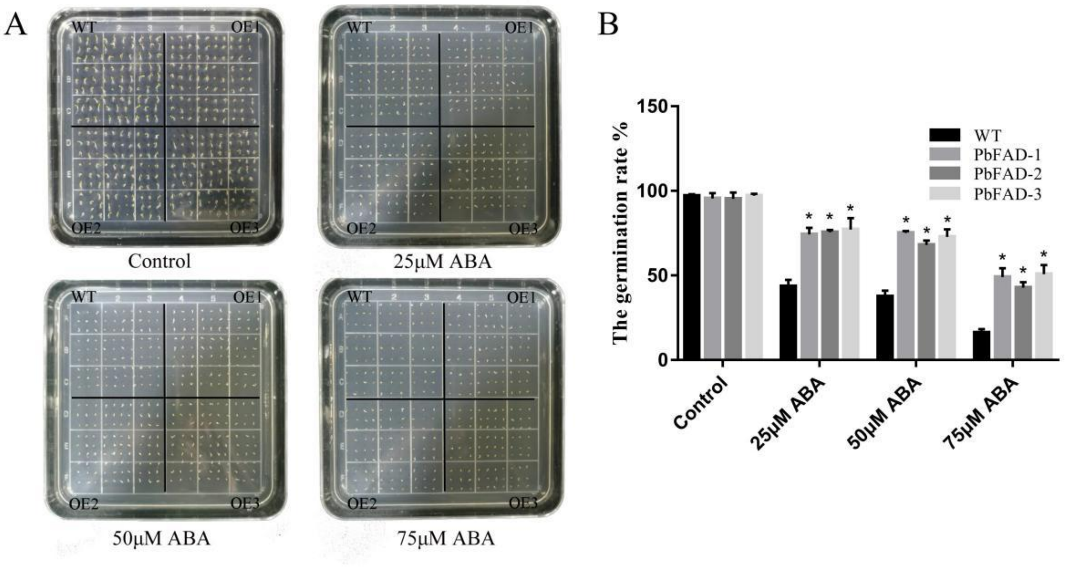

Fig. S8 Germination rate of *PbFAD3a* transgenic *Arabidopsis thaliana*. (A) Germination rate of transgenic *Arabidopsis thaliana* treated with ABA. (B) Statistical figure of germination rate after ABA treatment. (*p < 0.01). WT, wide type.
